# Nonlinear mixed-effects models for modeling *in vitro* drug response data to determine problematic cancer cell lines

**DOI:** 10.1038/s41598-019-50936-0

**Published:** 2019-10-08

**Authors:** Farnoosh Abbas-Aghababazadeh, Pengcheng Lu, Brooke L. Fridley

**Affiliations:** 1 Department of Biostatistics & Bioinformatics, Moffitt Cancer Center, Tampa, FL 33612 USA; 2 Department of Biostatistics, University of Kansas Medical Center, Kansas City, KS 66160 USA

**Keywords:** Cancer models, Cancer models, Statistics

## Abstract

Cancer cell lines (CCLs) have been widely used to study of cancer. Recent studies have called into question the reliability of data collected on CCLs. Hence, we set out to determine CCLs that tend to be overly sensitive or resistant to a majority of drugs utilizing a nonlinear mixed-effects (NLME) modeling framework. Using drug response data collected in the Cancer Cell Line Encyclopedia (CCLE) and the Genomics of Drug Sensitivity in Cancer (GDSC), we determined the optimal functional form for each drug. Then, a NLME model was fit to the drug response data, with the estimated random effects used to determine sensitive or resistant CCLs. Out of the roughly 500 CCLs studies from the CCLE, we found 17 cell lines to be overly sensitive or resistant to the studied drugs. In the GDSC, we found 15 out of the 990 CCLs to be excessively sensitive or resistant. These results can inform researchers in the selection of CCLs to include in drug studies. Additionally, this study illustrates the need for assessing the dose-response functional form and the use of NLME models to achieve more stable estimates of drug response parameters.

## Introduction

Over the past decades, the cancer cell lines (CCLs) have been widely used to study the biological processes in cancer, as well as *in vitro* drug screening for discovering and assessing the effectiveness of anticancer therapeutics^1^. Moreover, using *in vitro* CCL models to study cancer pharmacogenomics^2^ can be helpful to understand the resistance and sensitivity to therapy currently in use in cancer treatment, explore genomic factors associated with drug response, and develop more anticancer drugs^3^. Recently, two independent large-scale studies, the Cancer Cell Line Encyclopedia (CCLE)^4^ and the Genomics of Drug Sensitivity in Cancer (GDSC)^1,5^, were completed in which drug response information was collected on a number of therapeutic agents in addition to extensive molecular information (i.e., gene-expression profiles, mutational information). The use of large-scale drug studies on CCLs depends on the reliability and reproducibility of drug response assessments.

Despite the wide use of CCLs for drug response studies, inconsistency in drug-response data and poor concordance between mutational profiles of CCLs compare to patient tumors have been reported^6,7^. Multiple factors are likely to contribute to these observed inconsistencies in the drug-response data, including methodological and analytical challenges due to the differences in assay types, maximum tested drug concentration, range of tested drug concentrations, and drug sensitivity measurements employed by different studies^6,8^. In response, the authors of CCLE and GDSC reported a significantly better agreement for the differences between these two pharmacological data by incorporating both analytical and biological considerations^9^. However, discrepancies in the measured drug sensitivities still persist^10^. Furthermore, consistency can be achieved when biologically grounded analysis methods are incorporated using the standardization of assay methods and laboratory conditions^11,12^.

Testing of drug sensitivity has been a routine procedure in clinical and laboratory researches. Dose-response data collected on CCLs often are sigmoidal in shape and thus nonlinear logistic models are often used to model the data for each cell line individually^4,5,10,12,13^. Analysis of dose-response data typically focuses on the EC50 (i.e., half-maximum effective concentration)^14^, which is a description of shape of the dose-response curve at its midpoint, by which cell lines are often determined to be either sensitive or resistant. However, the dose-response curves can differ in other aspects, such as the slope of the curve or the area under the curve. Analyzing each cell line individually across a drug combination is highly problematic since the variation exhibited across the cell lines is ignored and this information could be harnessed inform the response profile of any single cell line (i.e., borrowing of information). Incorporating variation across all the cell lines in dose-response curve fitting can reduce noise and lead to more reliable inferences for any single CCL. Recently, the nonlinear mixed-effects (NLME) model has become an important approach to improve the accuracy of EC50 estimates (or similar parameters), through the borrowing of information across all CCLs^15,16^. Such models allow one to account for the repeated measures aspect in the data (i.e., correlation between measurements taken on the same cell lines) through the inclusion of random effects in model, along with fixed effects (i.e., dose of the drug) as included in traditional nonlinear regression models.

The objectives of this study are first to determine difference in model functional form (e.g., four-parameter logistic vs. three-parameter logistics) for drug-response data using data from the CCLE and GDSC. Then, for each cancer type and drug combination, a NLME model was fit to the drug-response data collected on the CCLs that had the same nonlinear functional form. Using the CCL specific estimated random effects from the NLME model, a set of cell lines were determined to be consistently sensitive or resistant to a large number of drugs. These findings can aid cancer researchers in the selection of cell lines to include in their experiments by eliminating CCLs that are always sensitive or resistant to drugs as results may not be generalizable. Moreover, this study illustrated the need for assessing model functional form for drug-response data and the ability to model all cell lines simultaneously using a NLME model to provide more stable estimates of drug response parameters.

## Methods and Materials

### Anticancer drug sensitivity studies

#### Cancer cell line encyclopedia (CCLE)

The drug sensitivity data available at http://broadinstitute.org/ccle was downloaded (February 2015) and processed. In total, the analysis consisted of *in vitro* drug response data for 8-point dose were collected on 24 compounds assessed on 504 cancer cell lines. Supplemental Fig. 1A represents the distribution of cancer types, where lung (18%) and hematopoietic and lymphoid tissue (14%) have the highest percentage of cell lines, while biliary tract, salivary gland, thyroid and prostate (<1%) have the lowest percentage of CCLs. Supplemental Fig. 1B demonstrates the distribution of cell lines under each drug across 23 cancer types represented in the CCLE.

#### Genomics of drug sensitivity in cancer (GDSC)

The drug sensitivity data are generated by the Cancer Genome Project at the Wellcome Trust Sanger Institute (WTSI) and the Center for Molecular Therapeutics at Massachusetts General Hospital using a collection of >1000 cell lines^1^. Drug sensitivity data included 213,605 drug-response series distributed over 990 cell lines and 265 drugs were downloaded from http://www.cancerrxgene.org (July 2016). The drug-response data were generated over 9 drug concentrations (2-fold dilution series) or 5 drug concentrations (4-fold dilution series). Supplemental Fig. 2A represents the distribution of cancer types, where 43% of cancer types have less than 1% cell lines, while non-small cell lung cancer (NSCLC) and small cell lung carcinoma have the most CCLs (6.5% of cell lines) in the study. Supplemental Fig. 2B presents the distribution of cell lines under each drug across 54 cancer types.

#### Overlap between the CCLE and GDSC

As both the CCLE and GDSC used existing CCLs, the two studies had a number of cell lines and drugs represented in both studies. In particular, dose-response data across 354 cell lines for 15 drugs were in common between the CCLE and GDSC. However, it should be noted that the two studies used different experimental protocols such as differences in the pharmacological assay types and the range of drug concentrations^6^.

### Dose-response models

Typically, an individual dose-response model is fitted to the *in vitro* dose response data for each CCL individually for a given drug. The shape of the dose-response curves vary between CCLs for a given drug (Supplemental Fig. 3A). From this nonlinear model, the EC50 is estimated, as either the inflection point of the sigmoidal curve (e.g., relative EC50 or IC50, the concentration that inhibits response by 50%) or the point at which the response is 50% (e.g., absolute EC50 or IC50)^14^ (Supplemental Fig. 3B). The absolute and relative EC50 will be the similar in the settings in which the top and bottom asymptotes of the dose-response curve are close to 100 and 0, respectively. However, the absolute EC50 is not estimable in all cases, while the relative EC50 is estimable in all cases, with the relative EC50 being recommended for use by Sebaugh (2011)^14^. Thus, we have chosen to use the relative EC50 which we refer to as the just the EC50 in the remainder of the paper. The most commonly used parametric nonlinear function for the observations on a given cell line is either a three-parameter (3P) or four-parameter (4P) logistic regression^13^. Although the functional form of the model remains the same for all cell lines (e.g., 4P logistic model), the parameter values vary from cell line to cell line.

Recently, nonlinear mixed-effects (NLME) model^15,17^ for repeated measures dose-response data have become popular due to their flexible covariance structure which allows for the joint modeling of multiple *in vitro* measurements taken off a set of CCLs. An advantage of using the NLME model is that both within-cell line and between-cell line variation are accounted to improve the statistical estimation of parameters. Moreover, the problem of extreme estimates caused by considering a single drug-cell line is reduced by using the NLME model where the information across cell lines is borrowed, with individual cell line parameters shrunk towards the population-level parameters^15–17^.

#### Nonlinear model

For a given drug, let *y*_*ij*_ represent the *j*^*th*^ dose-response at *n*_*i*_ drug doses, $j=1,2,\ldots ,{n}_{i}$, for the *i*^*th*^ cell line, $i=1,2,\ldots ,m$. Let *x*_*ij*_ denote the corresponding *j*^*th*^ drug dose assayed for the *i*^*th*^ cell line. The total number of measurements collected is equal to $N={\sum }_{i=1}^{m}{n}_{i}$. The relationship between the dose-response data and the drug doses can be described by the parametric nonlinear model,

1 $${y}_{ij}=f({x}_{ij},{{\boldsymbol{\beta }}}_{i})+{e}_{ij},$$

where *e*_*ij*_ is a random error term reflecting uncertainty in the response, given the *i*^*th*^ cell line. In Eq. (1), the regression function $f({x}_{ij},{{\boldsymbol{\beta }}}_{i})$ depends on ***β***_*i*_, the vector of *p* parameters, is referred to the functional form of the relationship and can take on numerous parameterizations. One commonly used functional form is the 4P logistic regression model,

2 $$f({x}_{ij},{{\boldsymbol{\beta }}}_{i})={\beta }_{1i}+\frac{{\beta }_{2i}-{\beta }_{1i}}{1+{e}^{[{\beta }_{4i}(log{x}_{ij}-{\beta }_{3i})]}},$$

with ${{\boldsymbol{\beta }}}_{i}=({\beta }_{1i},\ldots ,{\beta }_{4i})$. The parameters *β*_1*i*_ and *β*_2*i*_ represent responses at infinite and zero concentrations in the *i*^*th*^ cell line, respectively, *β*_3*i*_ is the logarithm of the concentration that gives a response midway between *β*_1*i*_ and *β*_2*i*_ (i.e., *log* EC50), and *β*_4*i*_ is the slope of the dose-response curve for the *i*^*th*^ cell line^18^. Collecting the errors for the *i*^*th*^ cell line into (*n*_*i*_ × 1) vector, ${{\boldsymbol{e}}}_{i}={({e}_{i1},{e}_{i2},\ldots ,{e}_{i{n}_{i}})}^{T}$, where the random error terms *e*_*ij*_ in Eq. (1) are assumed to be independent and identically normally distributed, ${{\boldsymbol{e}}}_{i} \sim N(0,{\sigma }_{i}^{2}{{\boldsymbol{I}}}_{{n}_{i}\times {n}_{i}})$. Under the classical assumption of normally distributed response values, the estimation of parameters in the 4P logistic regression model simplifies to nonlinear least squares approach, where the nonlinear least squares estimates are obtained by minimizing the weighted residual sum of squares^18^. However, in this study all responses are weighted equally. A Nelder-Mead derivative-free optimization algorithm is used to minimize the sum of squares of the residuals^19^.

Choice of the 4P logistic regression model is fairly typical in dose-response data analysis, while for some dose-response data, the 4P logistic regression model does not provide an adequate fit. In these cases, often a 3P logistic regression model is used by setting *β*_1*i*_ = 0 in Eq. (2). The nonlinear 3P and 4P logistic regression models, described in Eqs (1) and (2), can be fit using *drc* package in R^18^. Due to the behavior of the data, it may happen that neither 4P and nor 3P logistic regression models provide an adequate fit to the data. In such a case, other linear or nonlinear models can be fit to the data. For the analysis of the CCL data collected within the CCLE and GDSC, 4P logistic, 3P logistic, and a linear model (LM) were fit with models assessed with the Akaike’s information criterion (AIC)^20–22^ to account for the complexity of the model (i.e., the number of parameters in the model). The best fitting model was then determined for each CCL drug combination. Then, for each cancer site and drug combination, the model that fit most of the CCLs the best was selected as the best fitting model.

#### Nonlinear mixed-effects model

NLME model that is a generalization of both the linear mixed-effects model and the standard nonlinear fixed-effects model^15,17^. To model the drug response data for all CCLs for a given drug simultaneously, a NLME model is considered where the function form was either 3P or 4P logistic regression model. Although, the form of regression function *f* is common to all CCL dose-response data for a given drug, the parameter vector ***β***_*i*_ may vary across CCLs. Variation among different cell lines is accounted for through the cell line regression parameters where a model for the dependence of ***β***_*i*_ on random components is taken into account. This can be accomplished with a hierarchical model framework by adding the following second-level model to the nonlinear model outlined in Eqs (1) and (2). Let the between cell line variation be modeled through ***β***_*i*_ as

3 $${{\boldsymbol{\beta }}}_{i}={{\boldsymbol{A}}}_{i}{{\boldsymbol{\beta }}}_{0}+{{\boldsymbol{B}}}_{i}{{\boldsymbol{b}}}_{i},$$

where ***β***_0_ is (*r* × 1) vector of fixed-effects parameters representing the parameter values for the population, ***b***_*i*_ is a (*q* × 1) vector of random effects, and ***A***_*i*_ (*p* × *r*) and ***B***_*i*_ (*p* × *q*) are the design matrices associated with the fixed-effects and the random effects, respectively. The within-cell variation in Eq. (1) is normally distributed, ${{\boldsymbol{e}}}_{i}|{{\boldsymbol{b}}}_{i} \sim N(0,\,{\sigma }_{i}^{2}{{\boldsymbol{I}}}_{{n}_{i}\times {n}_{i}})$, and the cell line-to cell line variation is ${{\boldsymbol{b}}}_{i} \sim N(0,{{\bf{D}}}_{{\rm{qxq}}})$. Here, for a given drug, the regression parameters refer to the EC50 and slope can vary from cell line to cell line, thus resulting in two random effects for each parameter (Supplemental Fig. 3A). Thus, for the case of a 4P logistic regression function, this results in the following matrices and vectors: ***A***_*i*_ = ***I***_4×4_, $${{\boldsymbol{B}}}_{i}={{\textstyle (}\begin{array}{cccc}0 & 0 & 1 & 0\\ 0 & 0 & 0 & 1\end{array}{\textstyle )}}^{T}$$, ${{\boldsymbol{\beta }}}_{0}={({\beta }_{01},\ldots ,{\beta }_{04})}^{T}$, and ${{\boldsymbol{b}}}_{i}={({b}_{3i},{b}_{4i})}^{T}$. A combination of least squares estimators for nonlinear fixed effects models and maximum likelihood estimators for linear mixed-effects models were proposed to estimate both fixed and random effects, and variance components ${\sigma }_{i}^{2}$ and **D**^15,23^. For the analysis of the CCL data within the NLME model framework, the *nlme* R package was utilized^24^.

As a different NLME model was fit for all cell lines of a given cancer type and drug, we standardize the estimated cell lines random effects for each drug to enable comparison across drugs. For CCLE and GDSC datasets, the Spearman’s rank correlation coefficient (*r*_*s*_) was used to compare both the estimates of EC50 from the single cell line model and the standardized random effect estimates (SREs) from the NLME. Similar to Haibe-Kains, Benjamin, *et al*.^6^, the qualitative descriptions of correlation values are defined as; poor (*r*_*s*_ < 0.5), fair (0.5 ≤ *r*_*s*_ ≤ 0.6), moderate (0.6 ≤ *r*_*s*_ ≤ 0.7), substantial (0.7 ≤ *r*_*s*_ ≤ 0.8), and perfect (0.8 ≤ *r*_*s*_).

### Outlier cell lines detection

For a given drug, we fit two nonlinear logistic regression models (3P or 4P logistic) and a linear model (LM) to the dose-response data for each cell line, with the best fitting model determined based on AIC (Supplemental Fig. 4). Then, for a given cancer site and drug, the functional form (3P or 4P logistic) that fits the majority of CCLs was then used in fitting a NLME model. Note that only the CCLs that fit this functional form were included in the NLME model. From the NLME model, the estimated random effect for the EC50 was utilized to identify the outlier cell lines, as described in the following section.

To determine the cell lines that are consistently sensitive or resistant to a number of drugs assayed in the CCLE and GCSC, and therefore potential cell lines to remove from future drug studies, the estimated random effects were examined for each cell line and drug combination determined from the NLME model, as outlined in previous section^25^. Two procedures are considered to identify the outlier CCLs using the SREs. The first approach is based on determining three types of outliers; Type I or “mild” outlier cell line if SREs fall between (−2.5, −1.65] or [1.65, 2.5); Type II or “moderate” outlier cell line if SREs fall between (−3, −2.5] or [2.5, 3); and Type III or “extreme” outlier cell line if SREs fall outside of ±3. CCLs with SREs fall between (−1.65, 1.65) are neutral. These outlier types (I-III) are defined based on the boundaries of 95%, 99.4%, and 99.9% confidence intervals, respectively. CCLs with SREs < −1.65 or SREs > 1.65 across greater than or equal to 20% of drugs are sensitive or resistant, respectively, while the rest of CCLs were considered neutral. The second approach we employed to determine problematic CCLs or outliers was based on the distribution of the SREs. CCLs with SREs < 0 or SREs > 0 across greater than or equal to 80% of drugs are sensitive or resistant, respectively, while the rest of CCLs are neutral.

## Results

### Assessment of functional form

#### Cancer cell line encyclopedia (CCLE)

**Figure 1:**
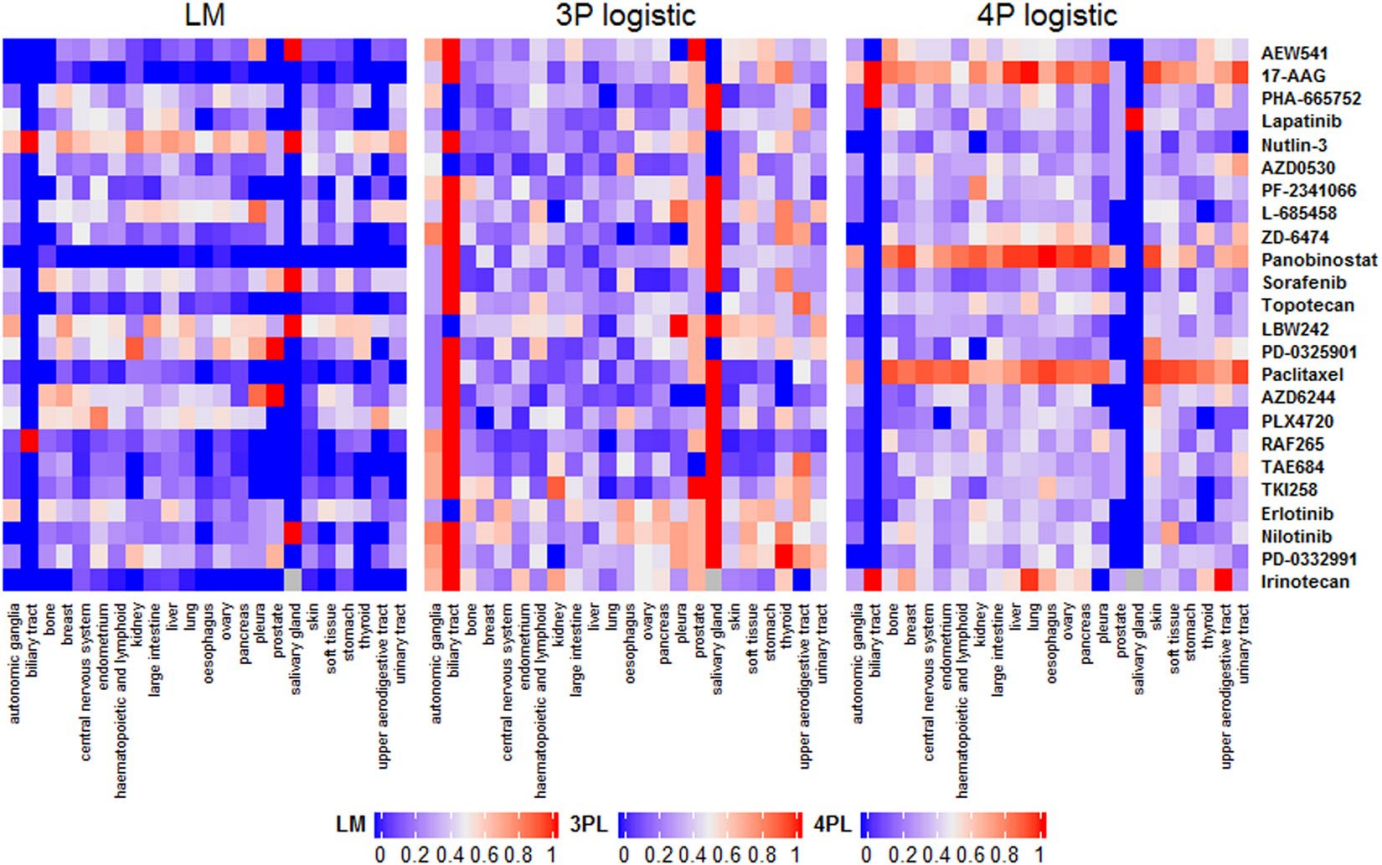
Heatmap of the proportion of cell lines where the best functional model using AIC was either the 3P logistic nonlinear model, 4P logistic nonlinear model, or the linear model (LM) computed across 24 drugs and 23 cancer types in the CCLE. The grey color represents situations where no drug treatment for a given cancer type.

Nonlinear models with functional forms of 4P and 3P logistic and a linear model (LM) were fit to the 497 CCLs in the CCLE, with best functional form based on AIC for each CCL and drug combination presented in Supplemental Tables 1 and 2. A summary of the model fits across cancer types and drugs was presented in Fig. 1. We observed that 42% of CCLs across all drugs and cancer types fit the 4P logistic model best, followed by 30% and 28% for 3P logistic model and LM, respectively. The 4P logistic model was consistently the best fitting model for the drugs Paclitaxel, Panobinostat, and 17-AAG (between 80–85% of CCLs). In contrast, the 3P logistic model fit the best for the majority of the drugs for the biliary track and salivary gland CCLs. Moreover, LM was the worst fitting model for most cancer types and drugs, while LM was the best fitting model for Nutlin-3 and LBW242 where LM best fitted model for around 55–60% of CCLs.

#### Genomics of drug sensitivity in cancer (GDSC)

**Figure 2:**
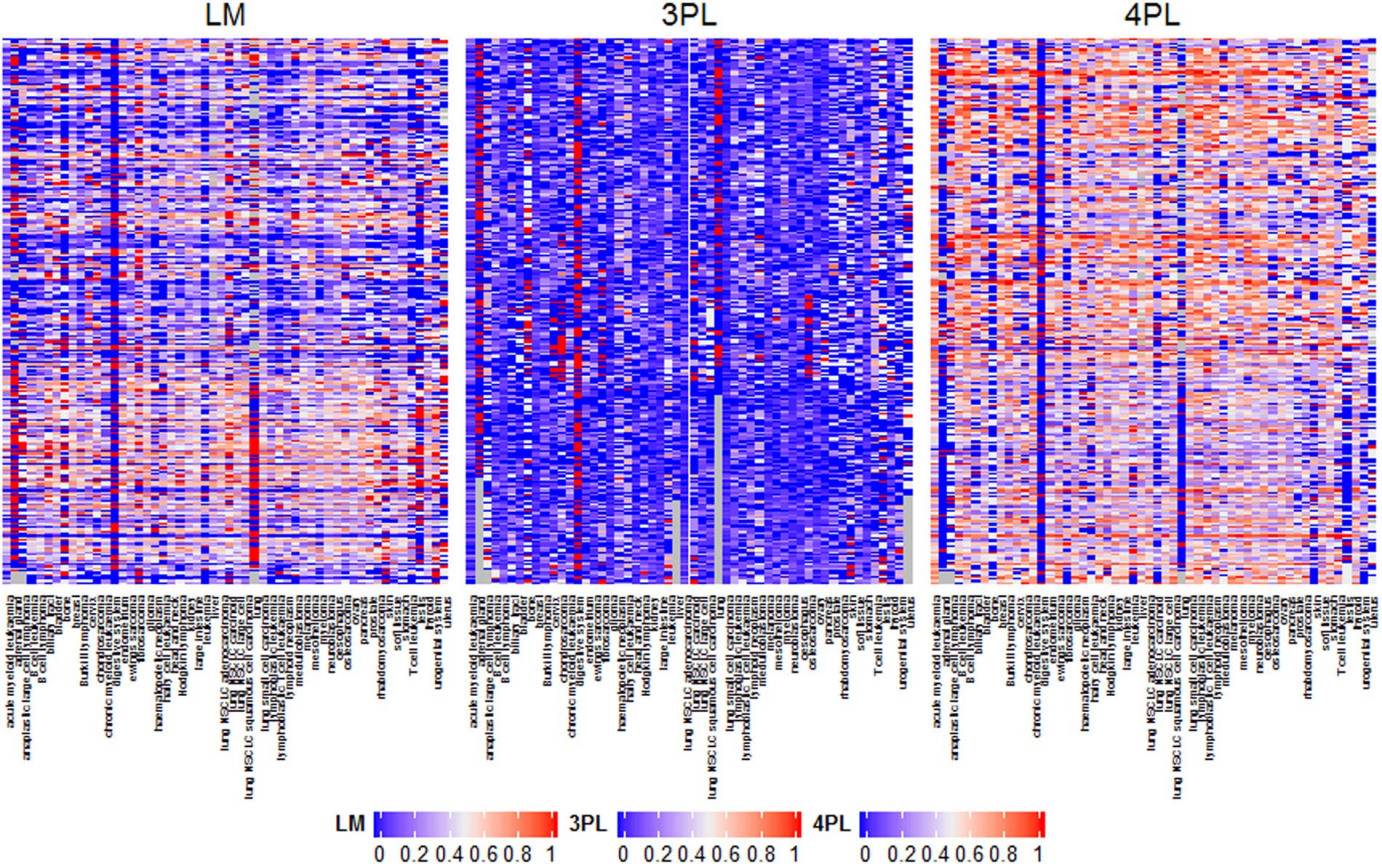
Heatmap of the proportion of cell lines where the best functional model using AIC was either the 3P logistic nonlinear model, 4P logistic nonlinear model, or the linear model (LM) computed across 265 drugs and 54 cancer types in the GDSC. The grey color represents situations where no drug treatment for a given cancer type.

Similar to the comparison of model fit statistics for the CCLE, we completed the assessment of functional forms (4P and 3P logistic, LM) for the 979 cell lines in the GDSC (see in Supplemental Tables 3 and 4). Figure 2 presents the proportion of fitted models for drugs across cancer types. We observed that the 4P logistic model was the best fit based on the AIC with 54% of CCLs across all drugs and cancer types fitting this functional form best, followed by 37% for LM and 10% for 3P logistic model. At the drug level (i.e., best fitting model per drug), the 4P logistic model was fitted for around 17% (44 out of 265) of drugs across the 70–85% CCLs. However, the 3P logistic model was the best fitting model for almost 8% (20 out of 265) of drugs across approximately 20–30% of CCLs. In contrast to the CCLE results, the LM fitted the best for 66 out of 265 drugs across the majority of CCLs (50–70%).

### Detection of outlier cell lines with NLME model

For a cancer type with more than 10 cell lines per drug, the functional form (3P or 4P logistic) which fit the majority of CCLs was used in fitting a NLME model defined in Eqs (1–3). Assessment of modeling assumptions was verified for 32 NLME models, as outlined and presented in the Supplemental Methods. In general, modeling assumptions were deemed to be valid for the majority of these selected drugs and cancer types, with a few drugs showing more “heavy tails” in the distribution than expected for a normal distribution. The estimated cell lines random effects (EC50) for each drug were standardized to enable comparison across drugs. Two proposed approaches were considered to identify the outlier cell lines using SREs for EC50 parameter.

#### Cancer cell line encyclopedia (CCLE)

**Figure 3:**
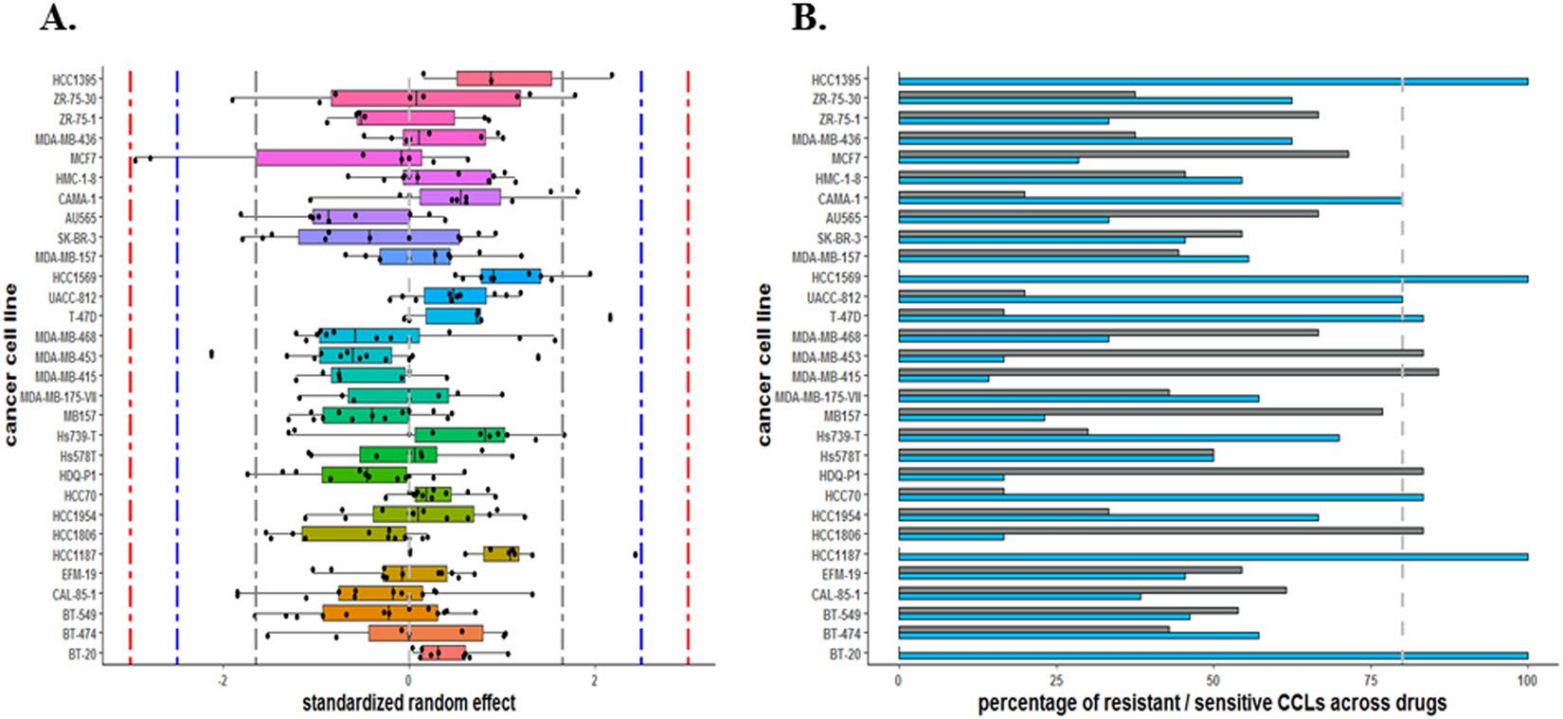
Standardized random effect estimates (SREs) for EC50 using a NLME model (via *nlme* package) for the breast cancer in CCLE. (**A**) Boxplots of the SREs to determine ‘outlier’ cell lines. Type I or ‘mild’ outlier cell line if SREs fall between (−2.5, −1.65] or [1.65, 2.5); Type II or ‘moderate’ outlier cell line if SREs fall between (−3, −2.5] or [2.5, 3); and Type III or ‘extreme’ outlier cell line if SREs fall outside of ±3. CCLs with SREs fall between (−1.65, 1.65) are neutral. (**B**) Percentage of breast CCLs with SREs < 0 or SREs > 0 across the drugs assessed. The ‘dash’ line shows the boundary 80%. The ‘blue’ and ‘grey’ bars represent the resistant and sensitive CCLs, respectively.

For the CCLE data, NLME modeling was completed for 15 out of 23 cancer types with more than 10 cell lines fitting the most common functional form for this cancer type and drug. As the NLME model was fit to cell lines that fit a 3P or 4P model for a given drug, all cell lines were not included in each model. However, all cell lines were included in a least 3 of the 24 NLME models (median = 12, range = 3 to 19). As an example, Fig. 3 shows the results from the NLME model to breast CCLs, where the cell line MCF7 has SREs between −3 and −2.5 indicating a moderately sensitive cell line for drugs AEW541 and TAE684. In addition, cell lines HCC1187, HCC1569, and HCC1395 had SREs between 1.65 and 2.5 (i.e., mild resistant) for 17-AAG, RAF265, and Panobinostat, respectively. The distribution of SREs for these three cell lines are also above 0 and thus look to be resistant to the majority of drugs. Supplementary Figure 5 presents the results for 454 SREs across cancer types and drugs in CCLE to visualize the outlier CCLs. Supplementary Table 5 contains the SREs and if the cell lines looks to be sensitive, resistance, or neutral for each drug across cancer types based on the value of SREs and the boundaries of 95%, 99.4%, and 99.9% confidence intervals. In addition to that, Supplementary Table 6 shows the proportion of sensitive or resistant based on a cut point of zero or ±1.65 across drugs, where 17% and 10% of CCLs are resistant (SREs > 0) and sensitive (SREs < 0) for greater than or equal to 80% of drugs, respectively. Moreover, 21 and 20 of CCLs (out of 454) are resistant (SREs > 1.65) and sensitive (SREs < −1.65) across greater than or equal to 20% of drugs, respectively. Table 1 represents the sensitive (or resistant) CCLs with both SREs < −1.65 and SREs < 0 (or SREs > 1.65 and SREs > 0) across greater than or equal to 20% and 80% of drugs, respectively. In particular, we found 7 cell lines; X42-MG-BA, PC-14, HUP-T4, ESS-1, JHH-5, TE-11, and A2780; to be overly sensitive and 10 cell lines OCI-LY10, HCC1395, EB2, SHP-77, EN, Toledo, KLE, JM1, EB1, and WM-115 to be overly resistant to many drugs. In addition, the majority of estimated genetic ancestry of outlier cell lines (9 out of 17) was reported as White/Caucasian, noting that the majority of the cell lines studied were from European ancestry^26^.

**Table 1:** Top sensitive (SREs < −1.65 and SREs < 0) and resistant (SREs > 1.65 and SREs > 0) cell lines across greater than or equal to 20% and 80% of drugs, respectively, in CCLE drug response data.

Cell Line	Cancer	Proportion (SREs < −1.65)	Proportion (SREs < 0)	Proportion (SREs > 1.65)	Proportion (SREs > 0)	Outlier	Estimated Genetic Ancestry
*X42-MG-BA*	central nervous system	0.43	0.86	—	—	sensitive	Caucasian
*PC-14*	lung	0.25	1	—	—	sensitive	Japanese
*HUP-T4*	pancreas	0.25	0.92	—	—	sensitive	Japanese
*ESS-1*	endometrium	0.25	0.88	—	—	sensitive	Caucasian
*JHH-5*	liver	0.22	1	—	—	sensitive	Japanese
*TE-11*	oesophagus	0.20	0.90	—	—	sensitive	Japanese
*A2780*	ovary	0.20	0.87	—	—	sensitive	African
*OCI-LY10*	haematopoietic and lymphoid	—	—	0.40	0.80	resistant	Caucasian
*HCC1395*	breast	—	—	0.33	1	resistant	Caucasian
*EB2*	haematopoietic and lymphoid	—	—	0.33	1	resistant	African
*SHP-77*	lung	—	—	0.29	1	resistant	Caucasian
*EN*	endometrium	—	—	0.25	0.88	resistant	Japanese
*Toledo*	haematopoietic and lymphoid	—	—	0.22	0.88	resistant	Caucasian
*KLE*	endometrium	—	—	0.20	0.80	resistant	Caucasian
*JM1*	haematopoietic and lymphoid	—	—	0.20	0.90	resistant	Caucasian
*EB1*	haematopoietic and lymphoid	—	—	0.20	1	resistant	African
*WM-115*	skin	—	—	0.20	0.80	resistant	Caucasian

#### Genomics of drug sensitivity in cancer (GDSC)

Similar to analysis completed on the CCLE, we completed the identification of outlier cell lines in the GDSC across 29 out of 54 cancer types with more than 10 cell lines. Similar to the CCLE analyses, all GDSC cell lines were not included in each model. The median number of NLME models that the cell lines in GDSC were included in was 74 drugs out of 242 drugs (range 3 to 153 drugs). Supplemental Figure 6 represents the outlier cell lines, where the visualization boxplots are considered for 857 SREs across drugs and cancer types. In addition, Supplementary Table 7 shows the SREs for EC50 and type of sensitivity and resistance of CCLs for each drug across cancer types based on the confidence interval approach in GDSC. Moreover, Supplementary Table 8 represents the proportion of sensitive (i.e., SREs < −1.65 or SREs < 0) and resistant (SREs > 1.65 or SREs > 0) cell lines across drugs in GDSC, where 39 and 59 of CCLs (out of 857) are resistant (SREs > 0) and sensitive (SREs < 0) for greater than or equal to 80% of drugs, respectively. However, 7 and 16 of CCLs (out of 857) are resistant (SREs > 1.65) and sensitive (SREs < −1.65) across greater than or equal to 20% of drugs, respectively. Table 2 represents the sensitive (or resistant) CCLs with both SREs < −1.65 and SREs < 0 (or SREs > 1.65 and SREs > 0) across greater than or equal to 20% and 80% of drugs, respectively, in GDSC drug response data. In the GDSC, we observed 11 cell lines to be overly sensitive (P31-FUJ, EW-24, RC-K8, NCI-H2291, SK-MEL-31, ME-1, DMS-53, TT, SCH, COLO-783, and NCI-H1838) and 4 cell lines to be exceedingly resistant (PA-1, PSN1, DMS-273, and A549). Lastly, the majority of estimated ancestry outlier CCLs (10 out of 15) was from white/Caucasian origin^26^.

**Table 2:** Top sensitive (SREs < −1.65 and SREs < 0) and resistant (SREs > 1.65 and SREs > 0) cell lines across greater than or equal to 20% and 80% of drugs, respectively, in GDSC drug response data.

Cell Line	Cancer	Proportion (SREs < −1.65)	Proportion (SREs < 0)	Proportion (SREs > 1.65)	Proportion (SREs > 0)	Outlier	Estimated Genetic Ancestry
*P31-FUJ*	acute myeloid leukaemia	0.37	0.90	—	—	sensitive	Japanese
*EW-24*	ewings sarcoma	0.36	0.92	—	—	sensitive	Caucasian
*RC-K8*	B cell lymphoma	0.32	0.95	—	—	sensitive	Japanese
*NCI-H2291*	lung NSCLC adenocarcinoma	0.32	0.89	—	—	sensitive	Caucasian
*SK-MEL-31*	melanoma	0.26	0.87	—	—	sensitive	Caucasian
*ME-1*	acute myeloid leukaemia	0.26	0.87	—	—	sensitive	Japanese
*DMS-53*	lung small cell carcinoma	0.26	0.82	—	—	sensitive	Caucasian
*TT*	thyroid	0.25	0.83	—	—	sensitive	Caucasian
*SCH*	stomach	0.22	0.84	—	—	sensitive	Japanese
*COLO-783*	melanoma	0.21	0.93	—	—	sensitive	Caucasian
*NCI-H1838*	lung NSCLC adenocarcinoma	0.20	0.89	—	—	sensitive	Caucasian
*PA-1*	ovary	—	—	0.30	0.88	resistant	Caucasian
*PSN1*	pancreas	—	—	0.23	0.85	resistant	Japanese
*DMS-273*	lung small cell carcinoma	—	—	0.21	0.89	resistant	Caucasian
*A549*	lung NSCLC adenocarcinoma	—	—	0.20	0.90	resistant	Caucasian

#### Overlap between the CCLE and GDSC

For CCLE and GDSC drug response data, we compared the estimates of EC50 using the SREs by fitting a NLME model and nonlinear model (3P and 4P logistic) across 354 CCLs and 15 drugs. For each drug, the Spearman’s rank correlation between SREs and nonlinear estimates of EC50 were computed (Supplemental Fig. 7). In CCLE, we observed that two drugs produced substantial correlations between the nonlinear model estimate and then NLME SRE estimates (17-AAG with *r*_*s*_ = 0.75; PLX4720 with *r*_*s*_ = 0.74), along with moderate correlations for drugs TAE684 (*r*_*s*_ = 0.56) and AZD0530 (*r*_*s*_ = 0.50) (Supplementary Fig. 7A,C). In the GDSC, drugs 17-AAG (*r*_*s*_ = 0.67) and Paclitaxel (*r*_*s*_ = 0.64) were observed with moderate correlations and drug Sorafenib (*r*_*s*_ = 0.52) had fair correlation between estimates (Supplementary Fig. 7B,D).

**Figure 4:**
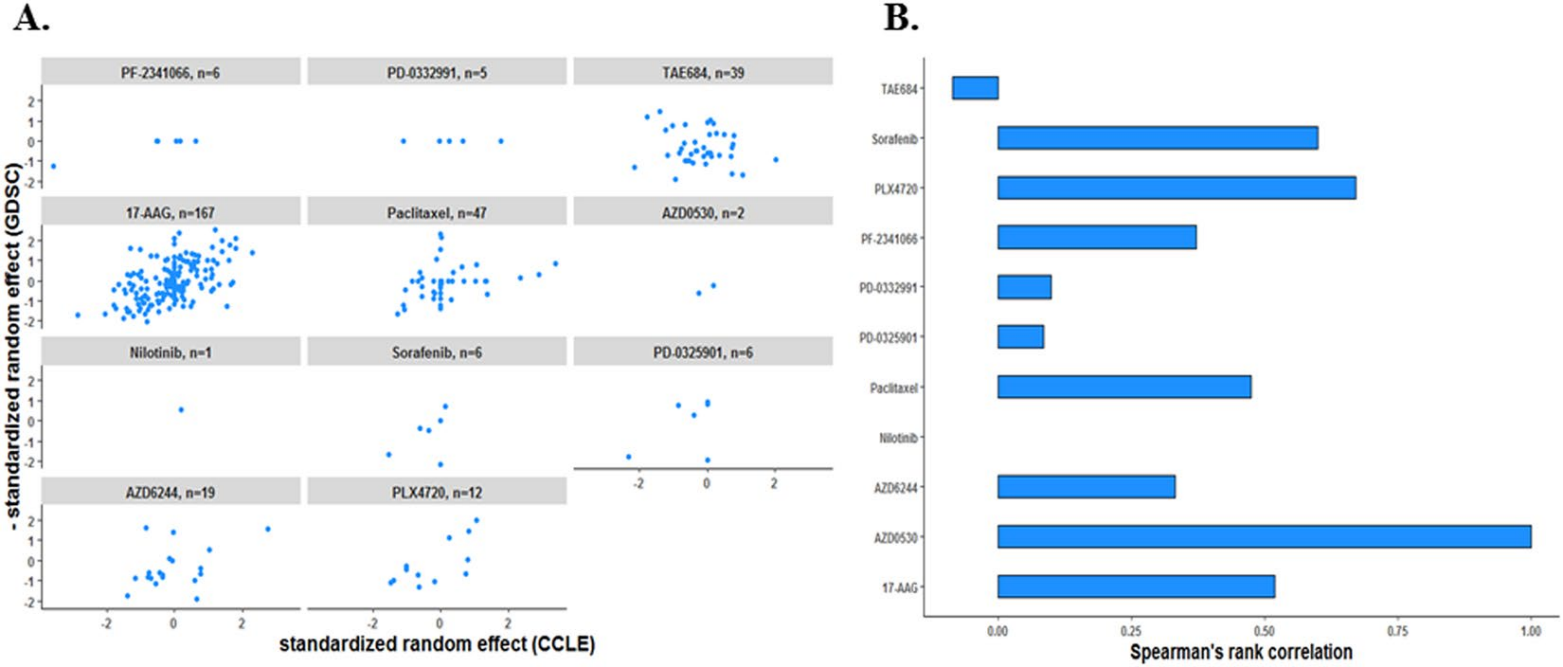
Consistency between drug response data using the standardized random effect estimates (SREs) across 196 CCLs and 11 drugs in both GDSC and CCLE: (**A**) Scatterplots displaying the relationship between the SREs from a NLME model (via *nlme* package) for the CCLE (x-axis) and the GDSC (y-axis). (**B**) Bar plot representing the Spearman’s rank correlation coefficient between the SREs from the CCLE and GDSC cell lines and drugs in common.

We then assessed the consistency between drug response data using SREs for EC50 across 196 CCLs and 11 drugs in both GDSC and CCLE, while the two studies used different experimental protocols^6,8,10–12^ (Fig. 4). We observed a single drug PLX4720 with moderate correlation (*r*_*s*_ = 0.67), and two drugs with fair correlations (17-AAG with *r*_*s*_ = 0.51; Paclitaxel with *r*_*s*_ = 0.50) between studies. The majority of drugs (7 out of 11) yielded poor concordance (*r*_*s*_ < 0.50).

## Discussion

The goals of this study were to first determine if there was a common functional form for *in vitro* drug response data generated on cell lines and second to determine using a nonlinear mixed-effects model cell lines that appear to be sensitive or resistant to a majority of the drug tested in either the CCLE or the GDSC studies. As the model for curve fitting has a direct impact on the measurement of drug potency (e.g., EC50), we compared the appropriateness of the 4P logistic, 3P logistic, and linear models for the 497 cell lines and 24 drugs in the CCLE and the 979 cell lines and 265 drugs in the GDSC. We observed that the best fitting dose-response relationships is often a 3 or 4 parameter logistic (3P logistic or 4P logistic) nonlinear model. However for some dose-response data, neither the 4P or 3P logistic models provided an adequate fit to the data. These results illustrate the need to assess the functional form for *in vitro* studies to ensure the most accurate and precise estimates of drug response, such as the EC50 or the area under the dose response curve. Further work is needed to assess other non-linear models besides the 3P and 4P logistic, such as the Cedergreen-Ritz-Streibig five-parameter model used in Moyer *et al*.^27^, for modeling drug-response data in the CCLE and GDSC.

The use of NLME model allows the joint modeling of all the CCL drug response data collectively with the ability to model the cell line-to cell line variation along with the within-cell line variation. Moreover, the flexible covariance structure allows both within-cell line and cell line-to-cell line variations are accounted to improve the statistical analysis. Such NLME model uses the information across the cell lines to adjust the extreme estimates caused by considering a single drug-cell line (i.e., borrowing of strength or information across cell lines). Therefore, a NLME model was used to detect sensitive or resistant cell lines with the SREs for the EC50 parameter using two approaches, one based on determination of outliers using the boundaries of confidence intervals and one based on the distribution of SREs for a CCL. We found 17 cell lines based on the CCLE data to be overly sensitive and resistant to many drugs (see Table 1). Similarly, we found 15 cell lines in the GDSC to be outliers in terms of either being sensitive or resistant to a majority of drugs (see Table 2). As passage information for the cell lines was not available in the dataset, we were not able to assess if this was a factor that might explain why these CCLs were outliers. However, we did not observe the same CCLs to be outliers between the CCLE and GDSC. Lastly, we assessed if the outlier cell lines had commonly mutated genes based on the Catalogue of Somatic Mutations in Cancer (COSMIC)^28^ (Supplementary Tables 9 and 10). In the CCLE, we found the following genes to be differentially mutated between either the sensitive or resistant cell lines vs the neutral cell lines, though none significant after adjustment for multiple testing: *KDR*, *PTEN*, *KRAS*, and *SPO11*. Moreover, in GDSC the following genes were differentially mutated with P value < 0.05: *RET*, *TNFAIP3*, *RAD21*, and STK11. Lastly, one gene, *KRAS*, was significantly differentially mutated between resistant and neutral CCLs in the GCSC (P value = 1.23e-08).

In conclusion, the results from this study can aid basic scientists in the selection of cell lines to include in experiments to ensure that results from the *in vitro* drug screens are generalizable. Additionally, this study illustrated the need for assessing functional form for the drug-response data and the ability to model all cell lines simultaneously using a NLME model to provide more accurate estimates of drug response parameters.

## Supplementary information

Supplemental Information

Supplemental Tables 1 - 10

## References

[1] Garnett MJ (2012). Systematic identification of genomic markers of drug sensitivity in cancer cells. Nature.

[2] Wang L, McLeod HL, Weinshilboum RM (2011). Genomics and drug response. New England Journal of Medicine.

[3] Gillet J-P (2011). Redefining the relevance of established cancer cell lines to the study of mechanisms of clinical anti-cancer drug resistance. Proceedings of the National Academy of Sciences.

[4] Barretina J (2012). The Cancer Cell Line Encyclopedia enables predictive modelling of anticancer drug sensitivity. Nature.

[5] Yang W (2012). Genomics of Drug Sensitivity in Cancer (GDSC): a resource for therapeutic biomarker discovery in cancer cells. Nucleic acids research.

[6] Haibe-Kains B (2013). Inconsistency in large pharmacogenomic studies. Nature.

[7] Domcke S, Sinha R, Levine DA, Sander C, Schultz N (2013). Evaluating cell lines as tumour models by comparison of genomic profiles. Nature communications.

[8] Weinstein JN, Lorenzi PL (2013). Cancer: discrepancies in drug sensitivity. Nature.

[9] Consortium CCLE, Consortium GODSIC (2015). Pharmacogenomic agreement between two cancer cell line data sets. Nature.

[10] Safikhani, Z. *et al*. Revisiting inconsistency in large pharmacogenomic studies. *F1000Research* **5** (2016). 10.12688/f1000research.9611.1 PMC5580432 28928933

[11] Mpindi JP (2016). Consistency in drug response profiling. Nature.

[12] Haverty PM (2016). Reproducible pharmacogenomic profiling of cancer cell line panels. Nature.

[13] Fallahi-Sichani M, Honarnejad S, Heiser LM, Gray JW, Sorger PK (2013). Metrics other than potency reveal systematic variation in responses to cancer drugs. Nature chemical biology.

[14] Sebaugh JL (2011). Guidelines for accurate EC50/IC50 estimation. Pharm Stat.

[15] Lindstrom, M. J. & Bates, D. M. Nonlinear mixed effects models for repeated measures data. *Biometrics*, 673–687 (1990). 2242409

[16] Vis DJ (2016). Multilevel models improve precision and speed of IC50 estimates. Pharmacogenomics.

[17] Davidian, M. *Nonlinear models for repeated measurement data*. (Routledge, 2017).

[18] Ritz C, Baty F, Streibig JC, Gerhard D (2015). Dose-response analysis using R. PLoS One.

[19] Nelder JA, Mead R (1965). A simplex method for function minimization. The computer journal.

[20] Akaike H (1987). Factor analysis and AIC. Psychometrika.

[21] Akaike, H. In *Selected papers of hirotugu akaike* 199–213 (Springer, 1998).

[22] Akaike, H. In *Selected Papers of Hirotugu Akaike* 215–222 (Springer, 1974).

[23] Lindstrom MJ, Bates DM (1988). Newton—Raphson and EM algorithms for linear mixed-effects models for repeated-measures data. Journal of the American Statistical Association.

[24] Pinheiro, J. *et al*. Package ‘nlme’. *Linear and Nonlinear Mixed Effects Models, version*, 3–1 (2017).

[25] Fellner WH (1986). Robust estimation of variance components. Technometrics.

[26] Dutil J, Chen Z, Monteiro AN, Teer JK, Eschrich SA (2019). An Interactive Resource to Probe Genetic Diversity and Estimated Ancestry in Cancer Cell Lines. Cancer Res.

[27] Moyer AM (2011). Acetaminophen-NAPQI hepatotoxicity: a cell line model system genome-wide association study. Toxicol Sci.

[28] Forbes SA (2014). COSMIC: exploring the world’s knowledge of somatic mutations in human cancer. Nucleic acids research.

