# Supplementary material for: Nonlinear mixed-effects models for modeling in vitro drug response data to determine problematic cancer cell lines
Source: Sci Rep. 2019 Oct 8;9:14421. doi: 10.1038/s41598-019-50936-0 (PMC6783462; doi:10.1038/s41598-019-50936-0)
Supplement: Supplementary file 1 — Supplemental Information [file 41598_2019_50936_MOESM1_ESM.pdf]

Nonlinear mixed-effects models for modeling *in vitro* drug response data to determine problematic cancer cell lines

Farnoosh Abbas-Aghababazadeh[1], Pengcheng Lu[2], and Brooke L. Fridley[1*]

[1]Department of Biostatistics & Bioinformatics, Moffitt Cancer Center, Tampa, FL, 33612, USA
[2]Department of Biostatistics, University of Kansas Medical Center, Kansas City, KS, 66160, USA

Supplemental Figures:

Supplemental Figure 1: CCLE drug response data: (**A**) Distribution of 23 cancer types and (**B**) distribution of 504 cell lines under 24 drugs across 23 cancer types.

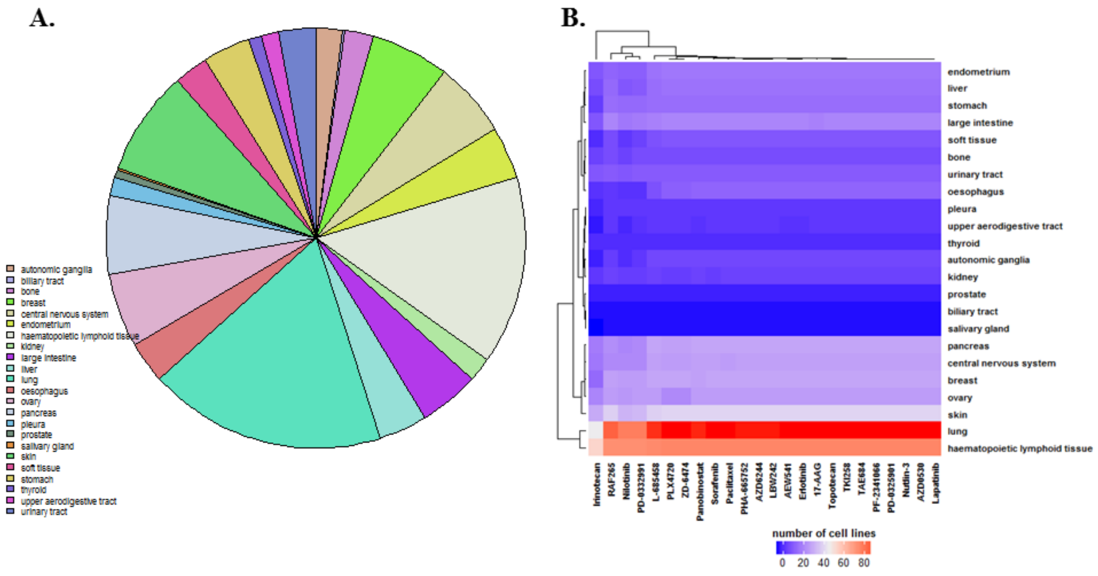

Supplemental Figure 2: GDSC drug response data: (**A**) Distribution of 54 cancer types and (**B**) distribution of 990 cell lines under 265 drugs across 54 cancer types.

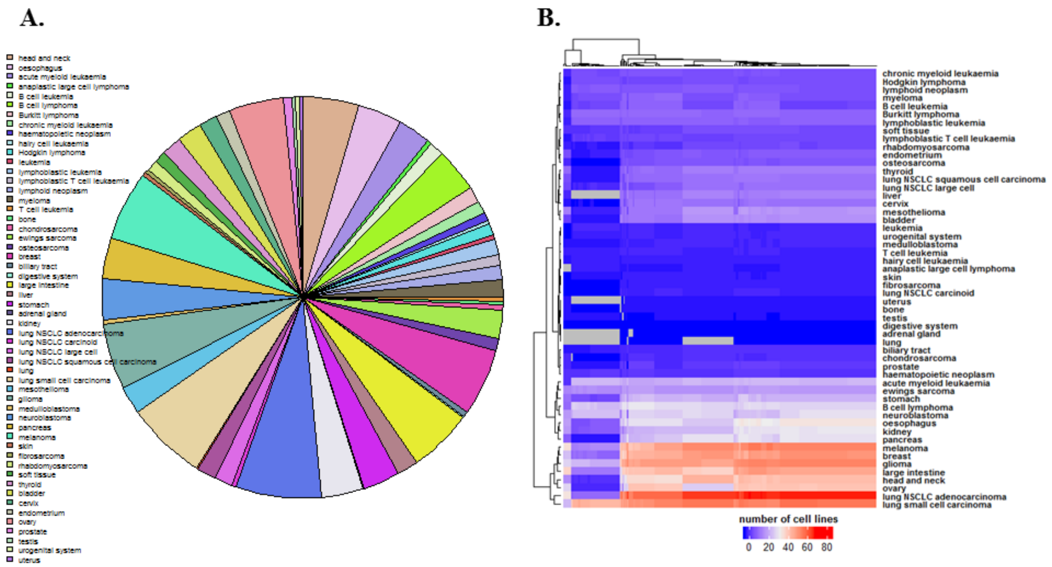

Supplemental Figure 3: (**A**) Pattern of drug response across the selected liver cell lines for drugs 17-AAG and Topotecan. (**B**) Compare the relative and absolute EC50 by fitting 4PL nonlinear model on the CCLE drug response data. Two ways of defining EC50 are; relative EC50 ‘dot’ line and absolute EC50 ‘dot-dashed’ line.

A.

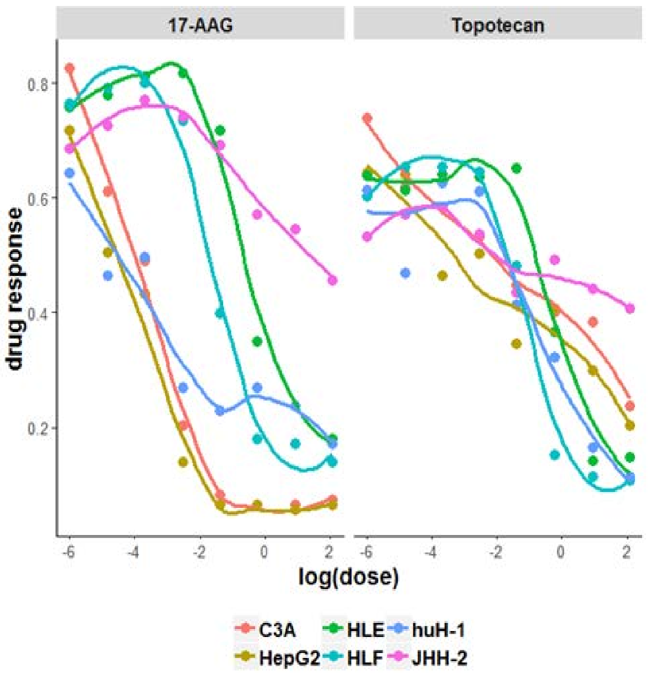

B.

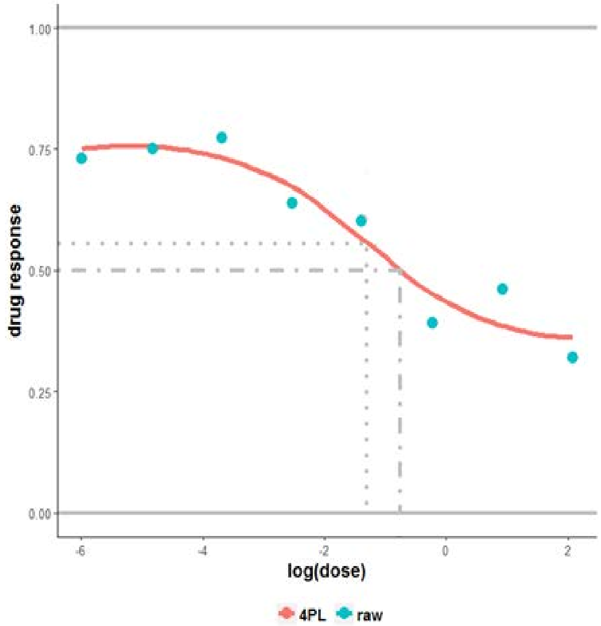

Supplemental Figure 4: Comparison of 3P and 4P logistic nonlinear (via *drc* package) and linear (LM) models using AIC for CCLE drug response data; (**A**) 4P logistics nonlinear model, (**B**) 3P logistics nonlinear model, and (**C**) LM has the minimum AIC values.

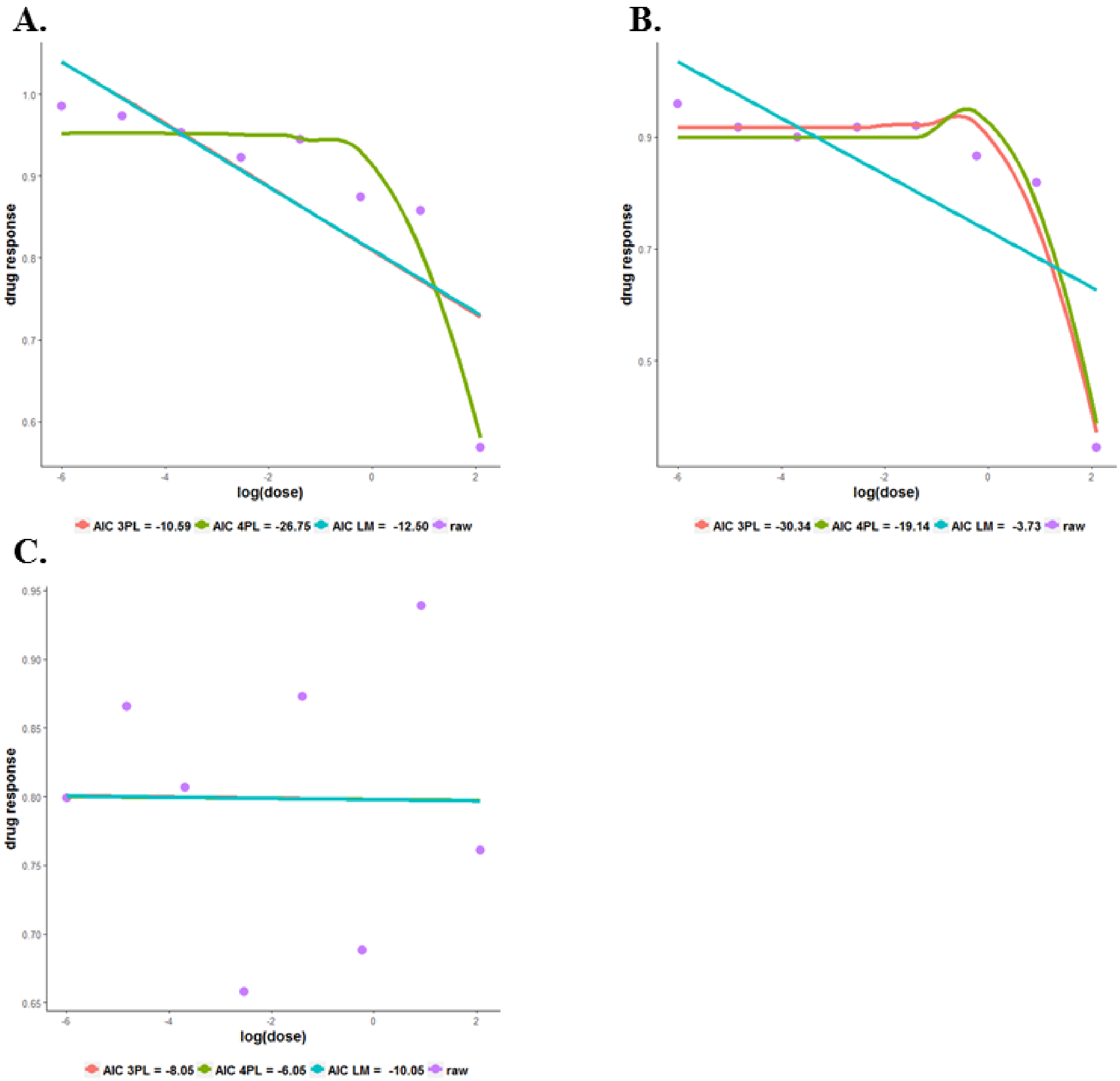

Supplemental Figure 5: Fitting a NLME model (via *nlme* package) for 15 out of 23 cancer types with more than 10 cell lines in CCLE. To determine the outlier cell lines, the standardized random effect estimates (SREs) are plotted under each cell line. Left panel: the vertical 'two-dash' lines show the boundary for the mild, moderate and extreme outlier CCLs. The 'dash' line shows the boundary zero. Right panel: percentage of CCLs with SREs < 0 and SREs > 0 across drugs. The 'dash' line shows the boundary 80%. The 'blue' and 'grey' bars represent the resistant and sensitive CCLs, respectively.

Bone

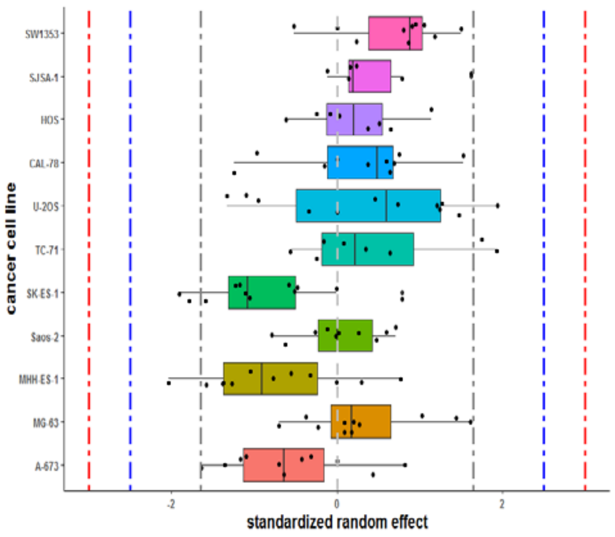

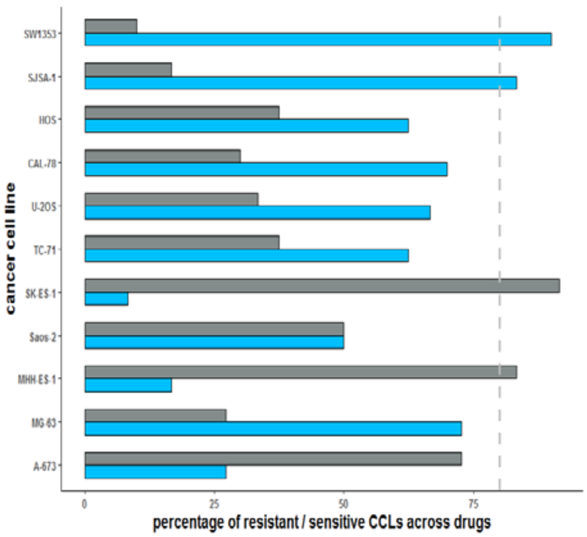

Central nervous system

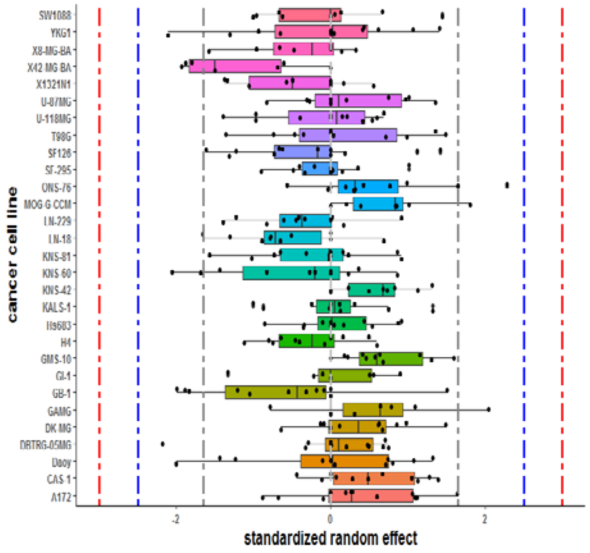

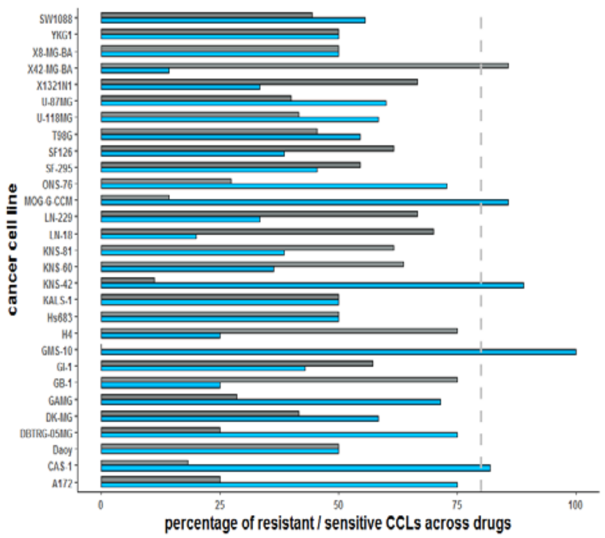

Endometrium

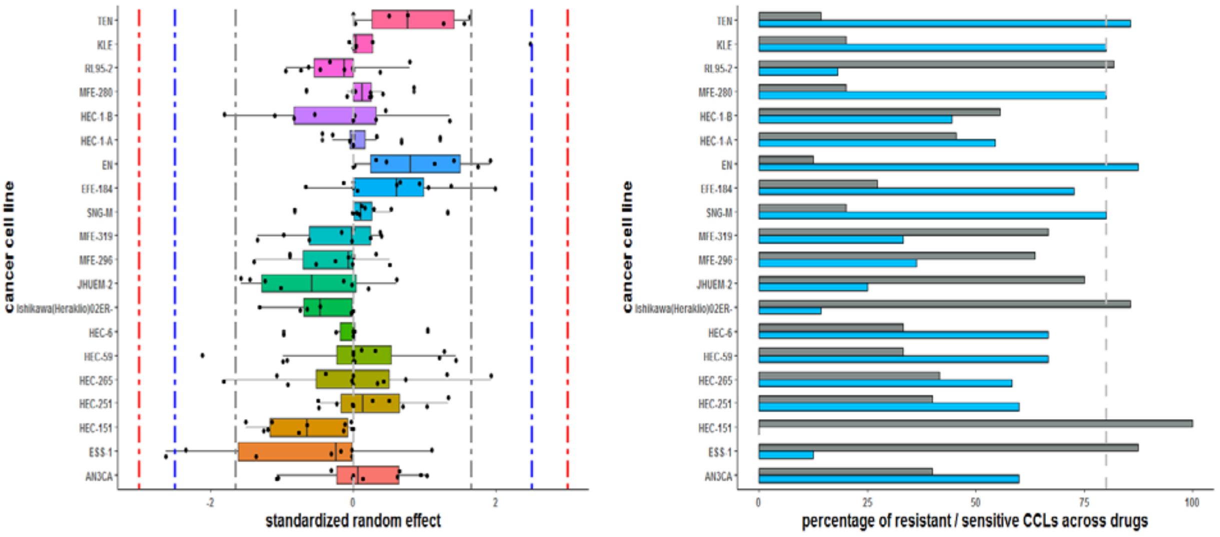

Haematopoietic and lymphoid

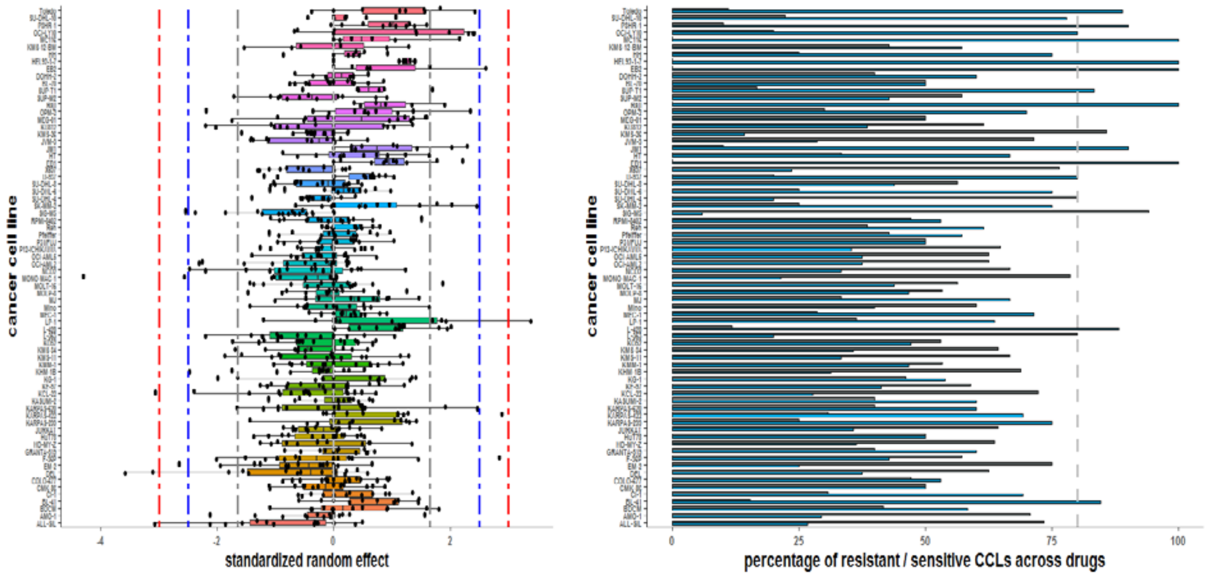

Large intestine

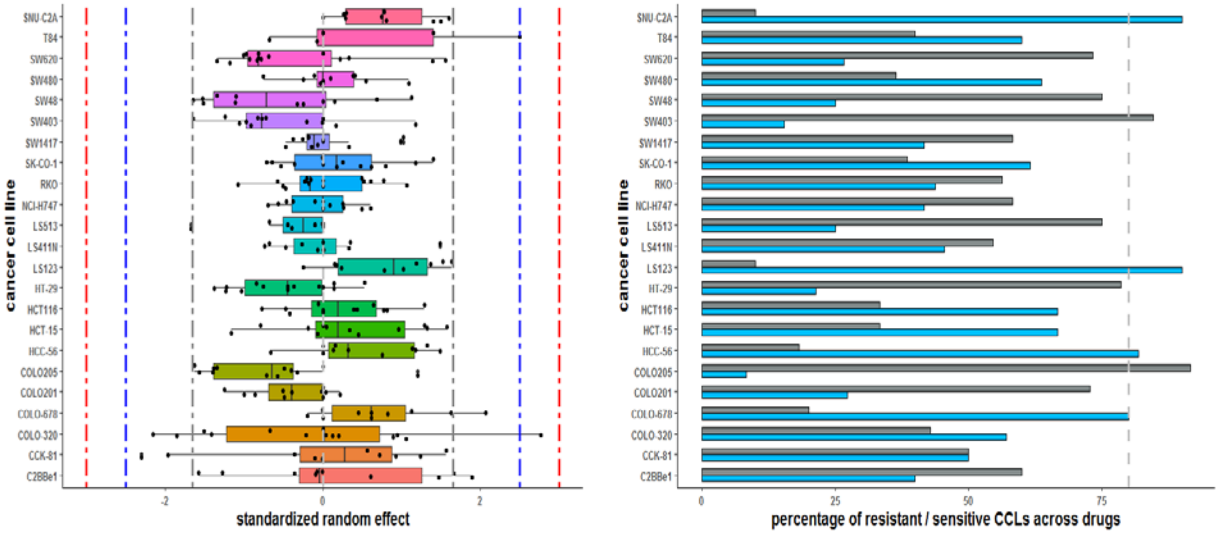

Liver

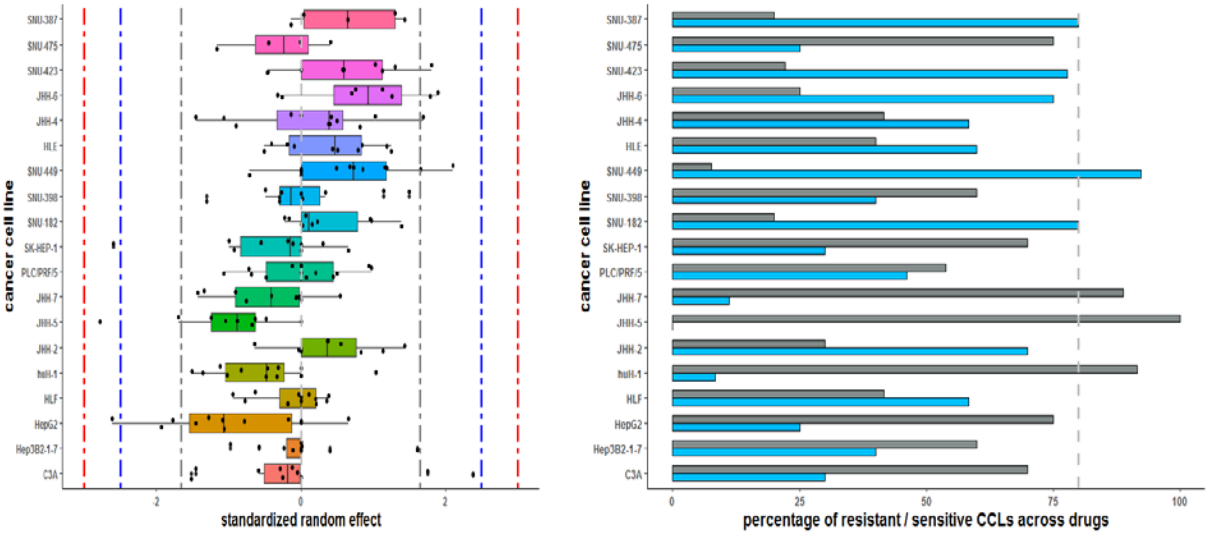

Lung

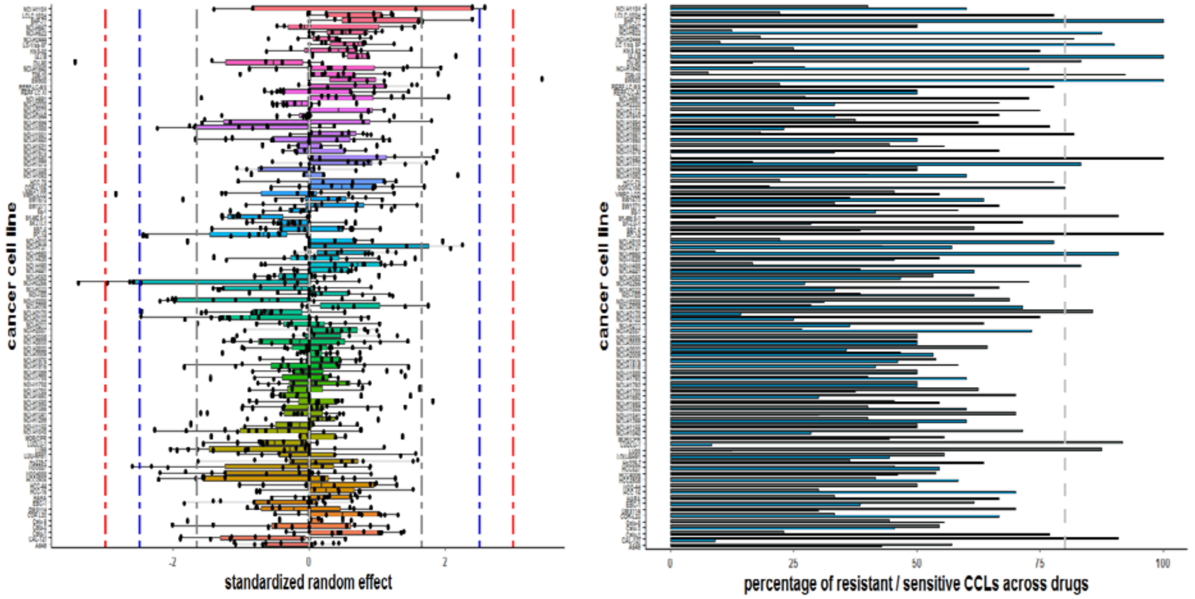

Oesophagus

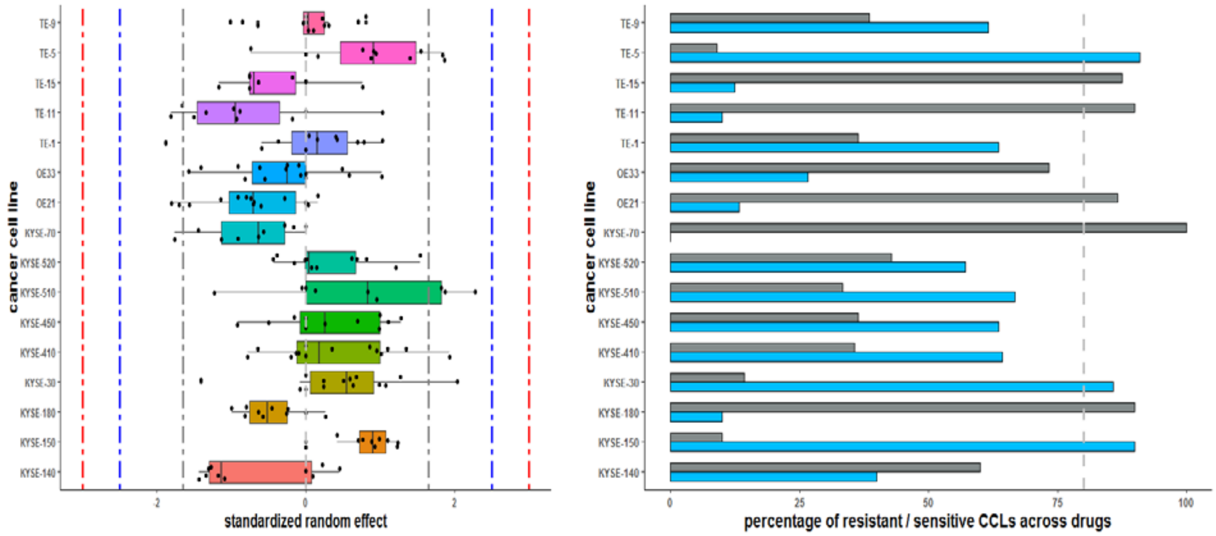

Ovary

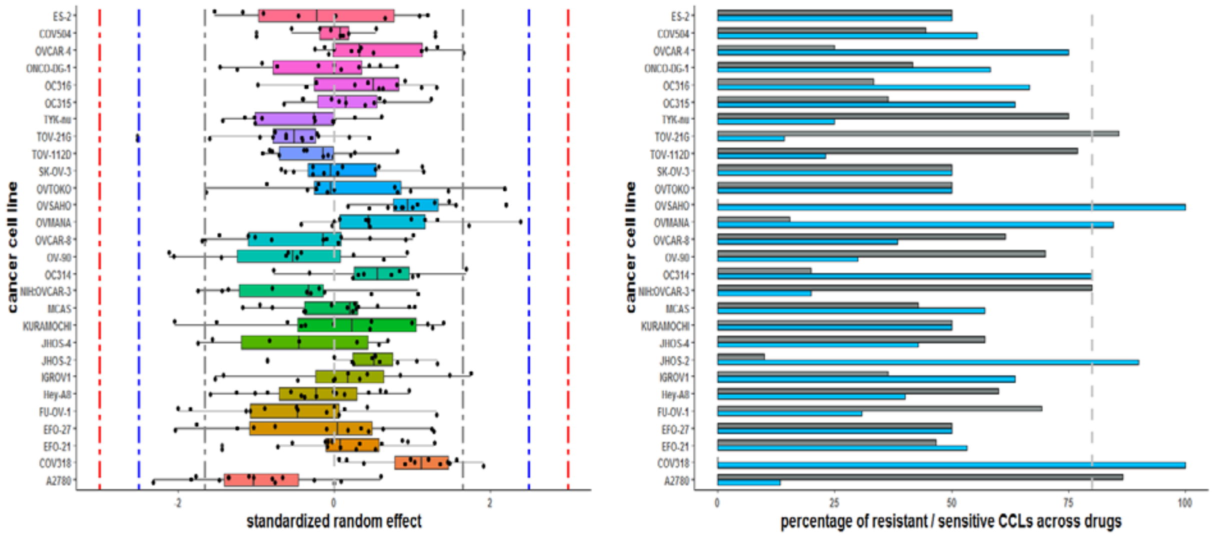

Pancreas

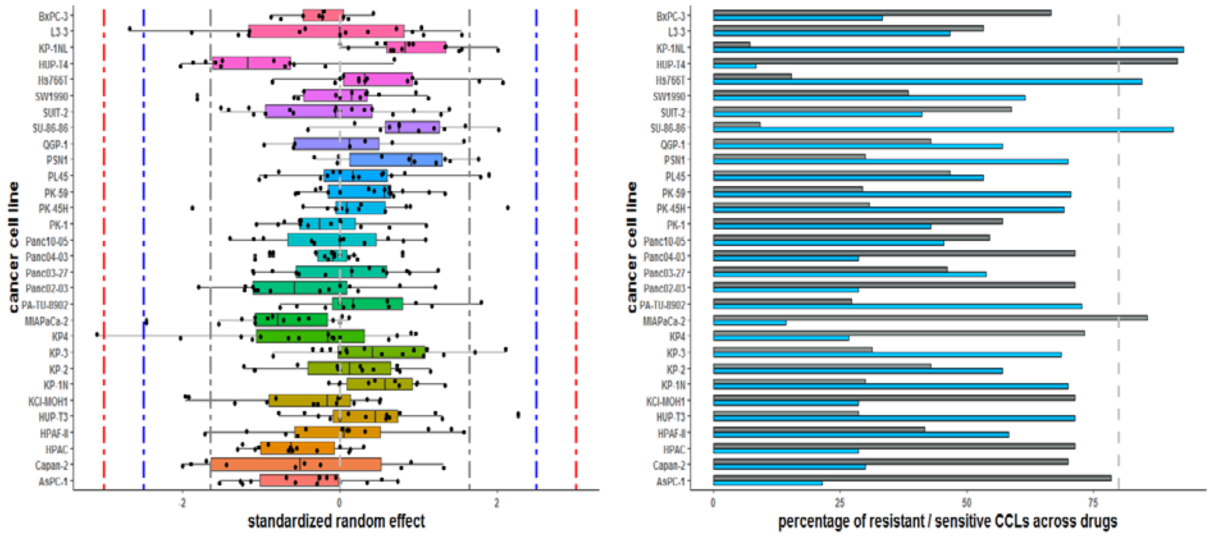

Skin

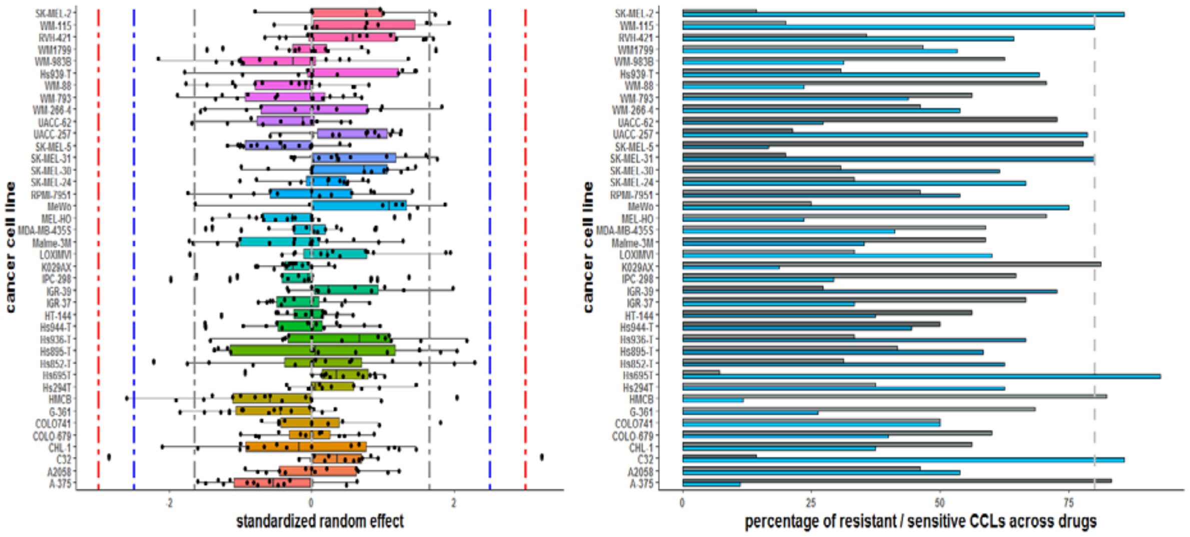

Soft tissue

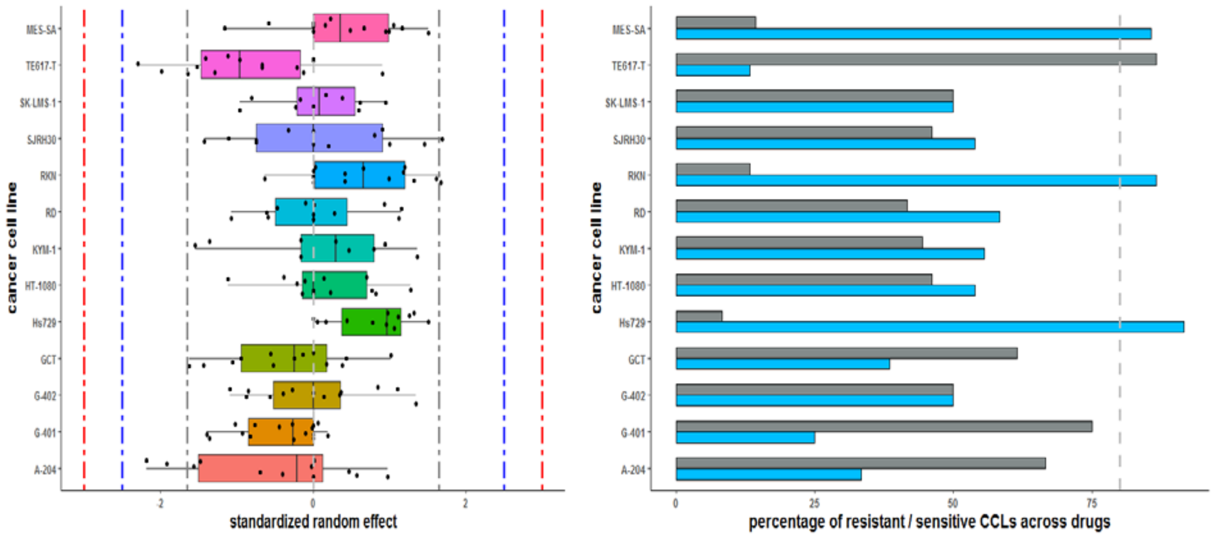

Stomach

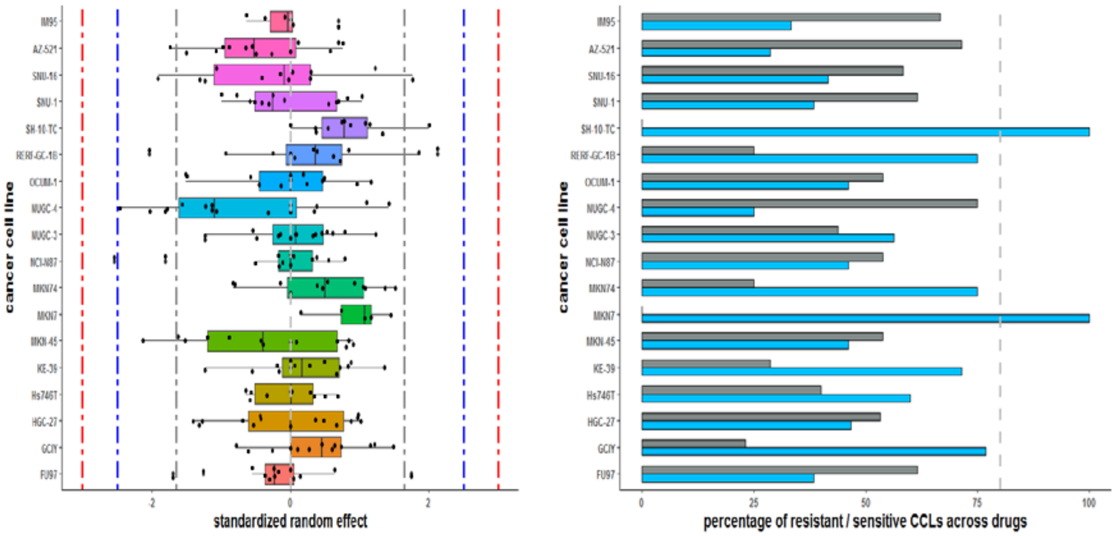

Urinary tract

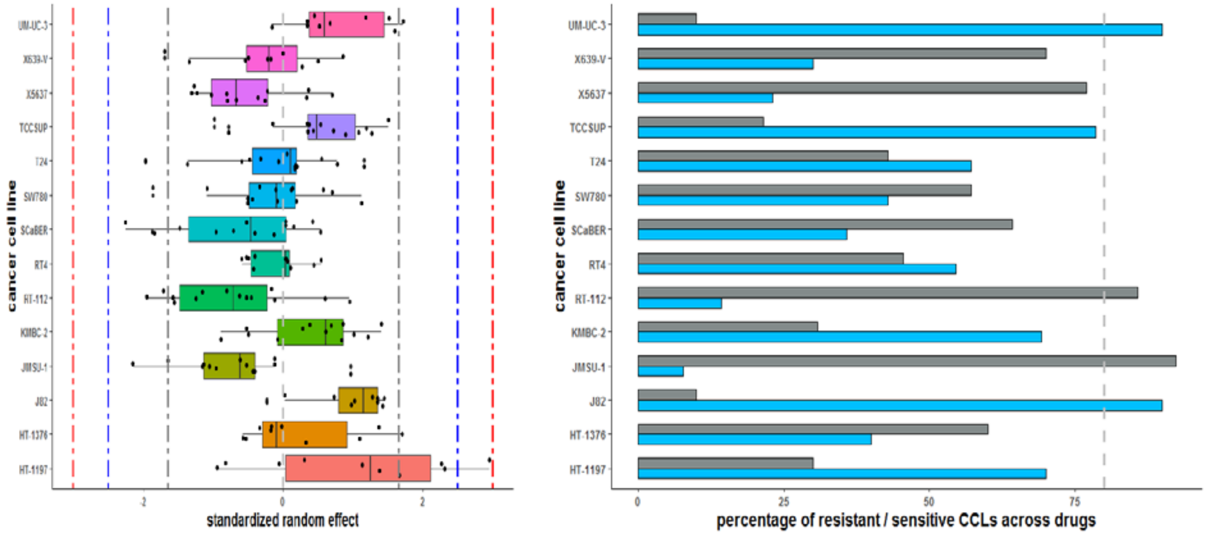

Supplemental Figure 6: Fitting a NLME model (via *nlme* package) for 29 out of 54 cancer types with more than 10 cell lines in GDSC. To determine the outlier cell lines, the standardized random effect estimates (SREs) are plotted under each cell line. Left panel: the vertical 'two-dash' lines show the boundary for types I, II, and III to get the mild, moderate and extreme outlier CCLs. The 'dash' line shows the boundary zero. Right panel: percentage of CCLs with SREs < 0 and SREs > 0 across drugs. The 'dash' line shows the boundary 80%. The 'blue' and 'grey' bars represent the resistant and sensitive CCLs, respectively.

Acute myeloid leukaemia

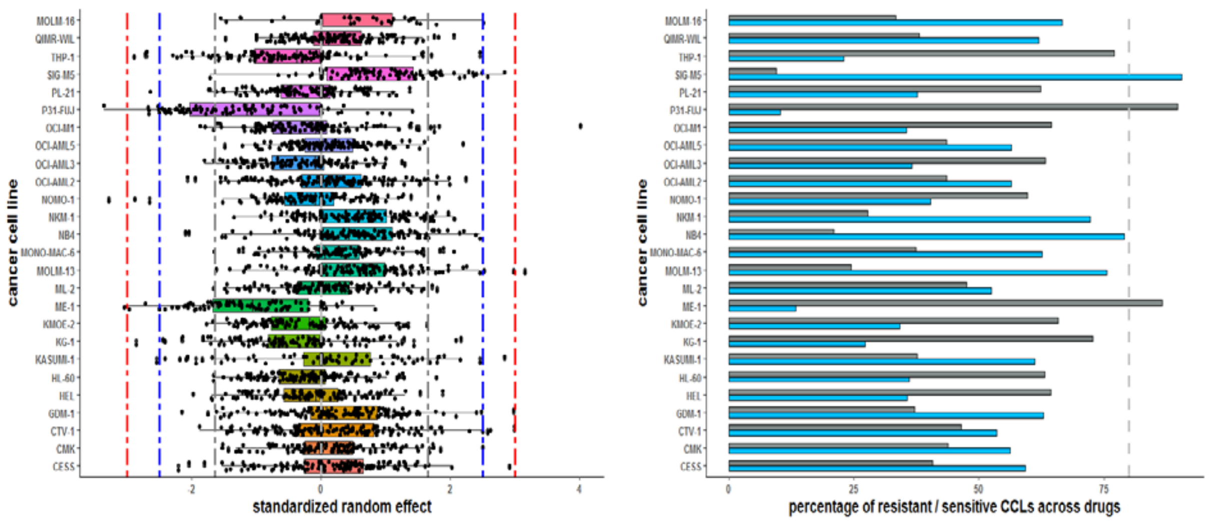

B cell leukemia

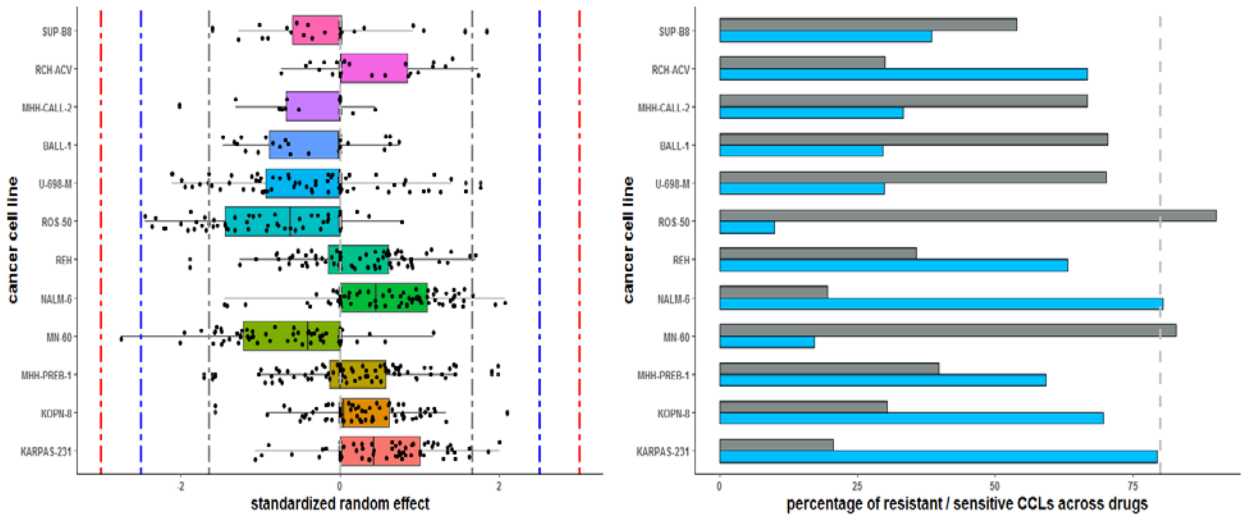

B cell lymphoma

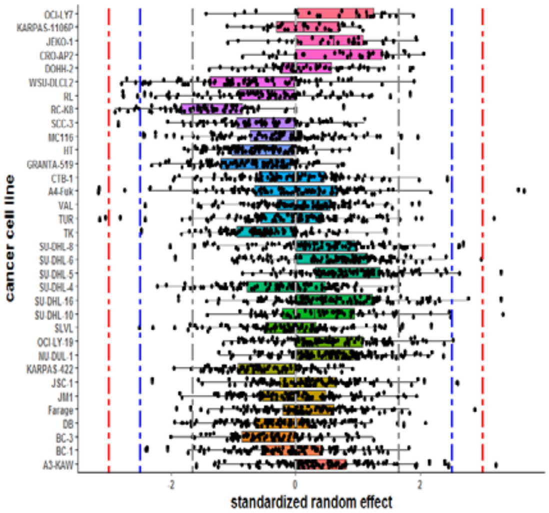

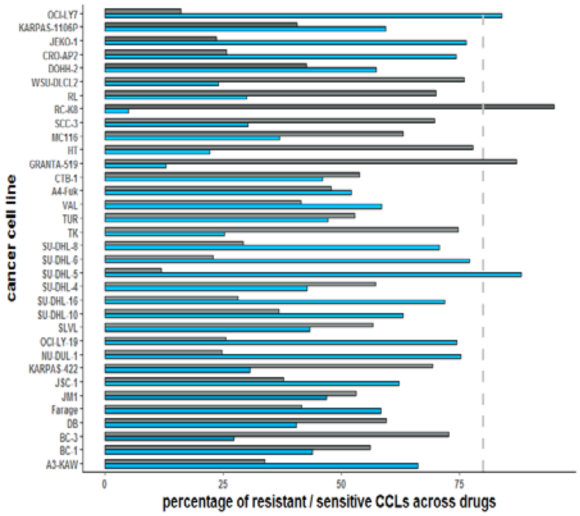

Bladder

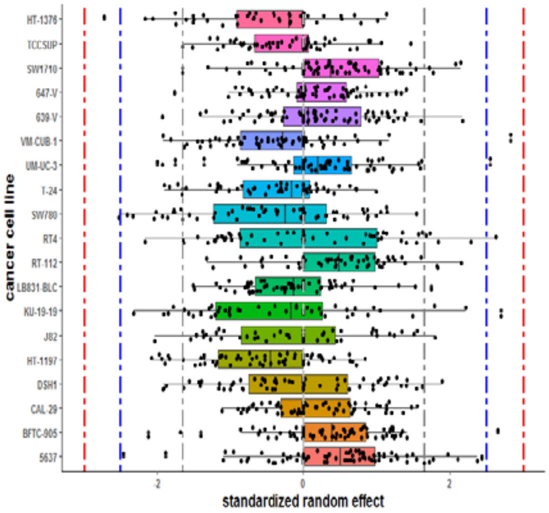

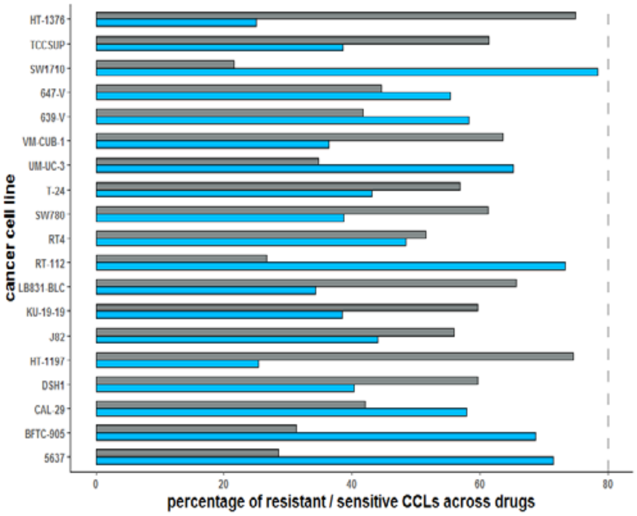

Breast

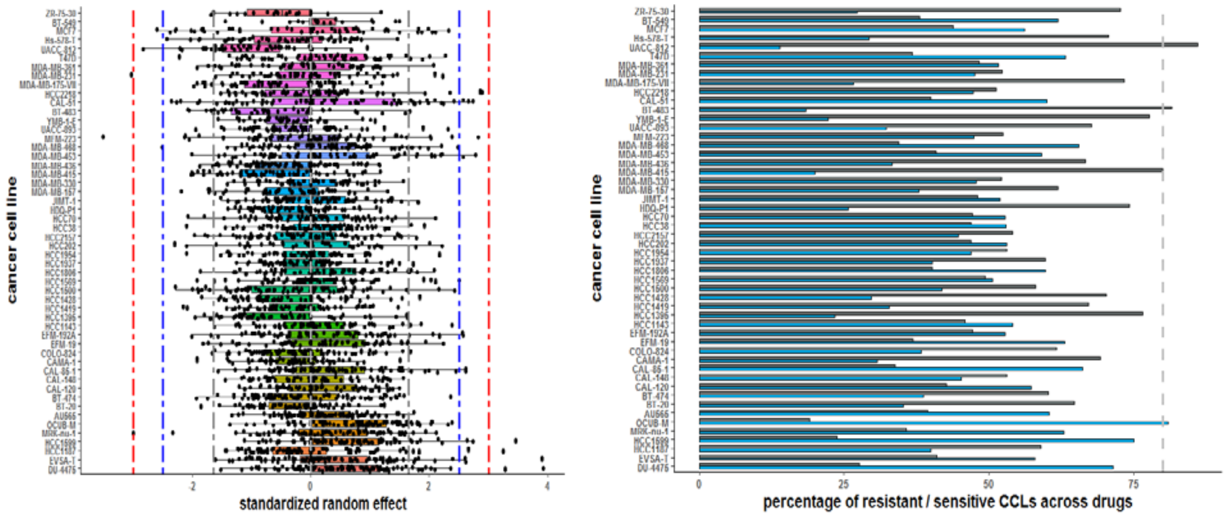

Burkitt lymphoma

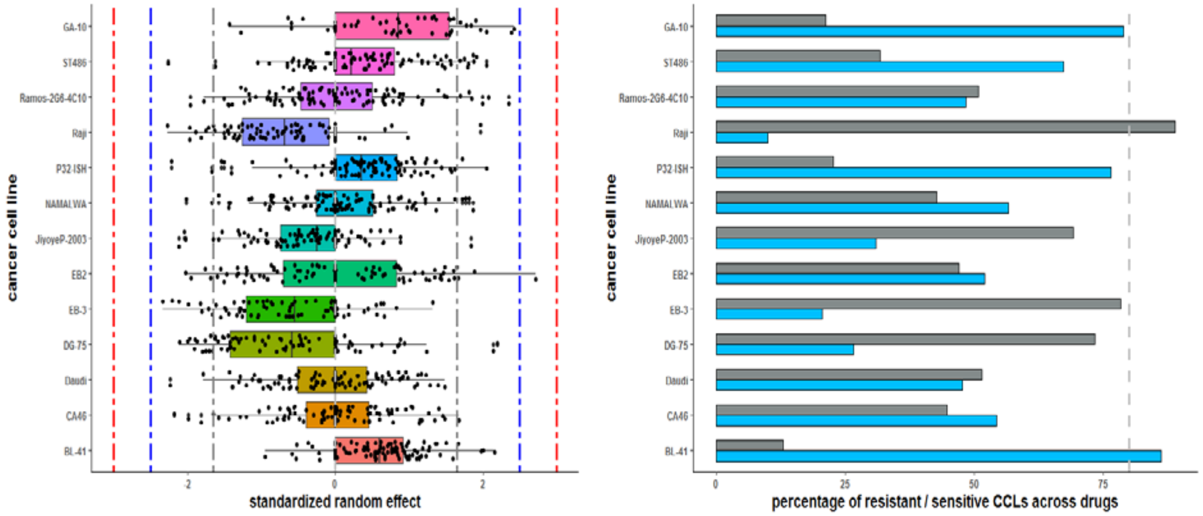

Cervix

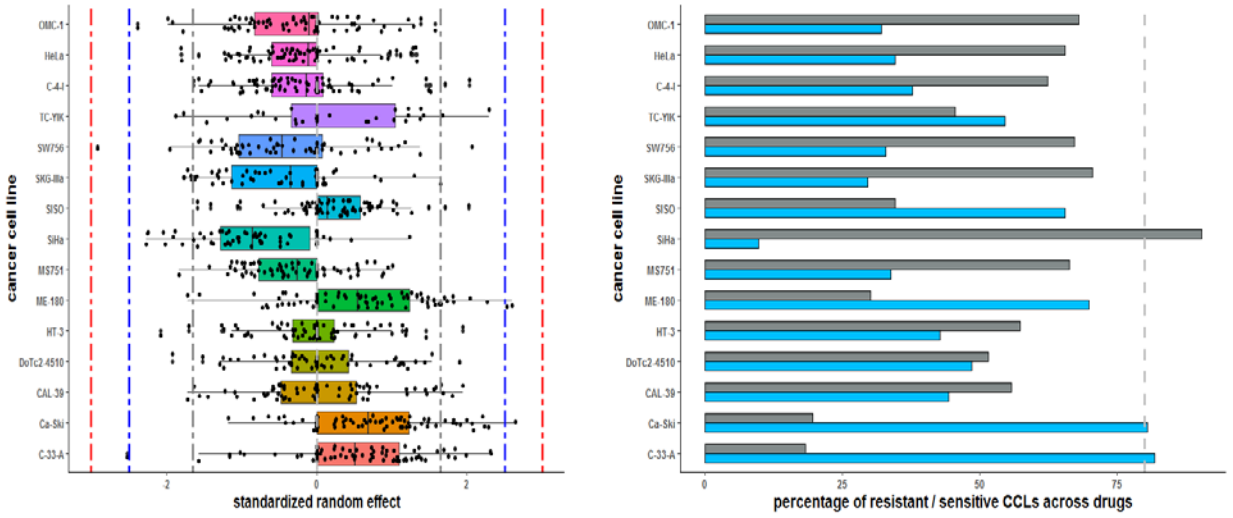

Endometrium

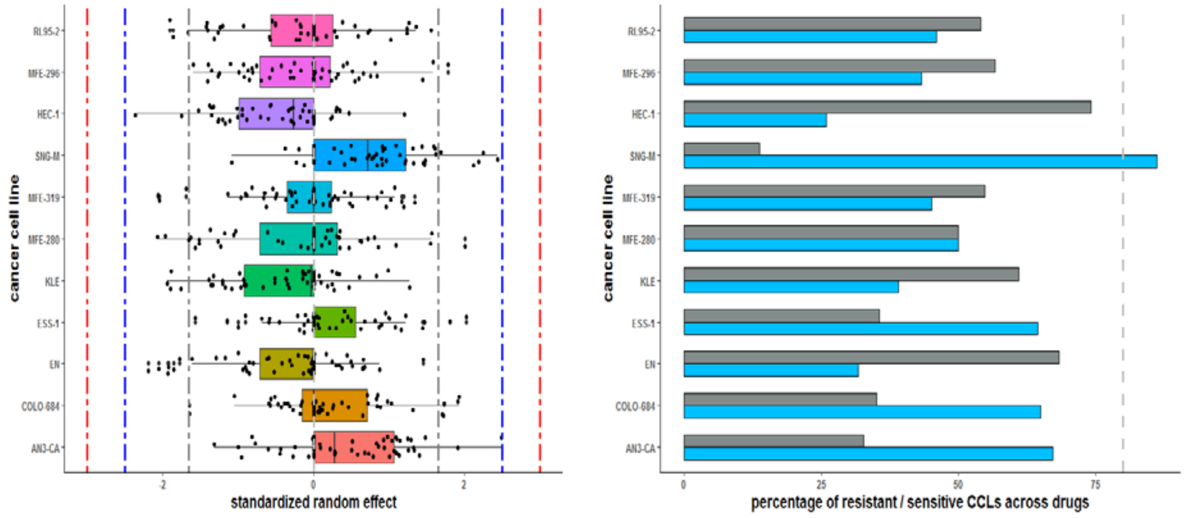

Ewings sarcoma

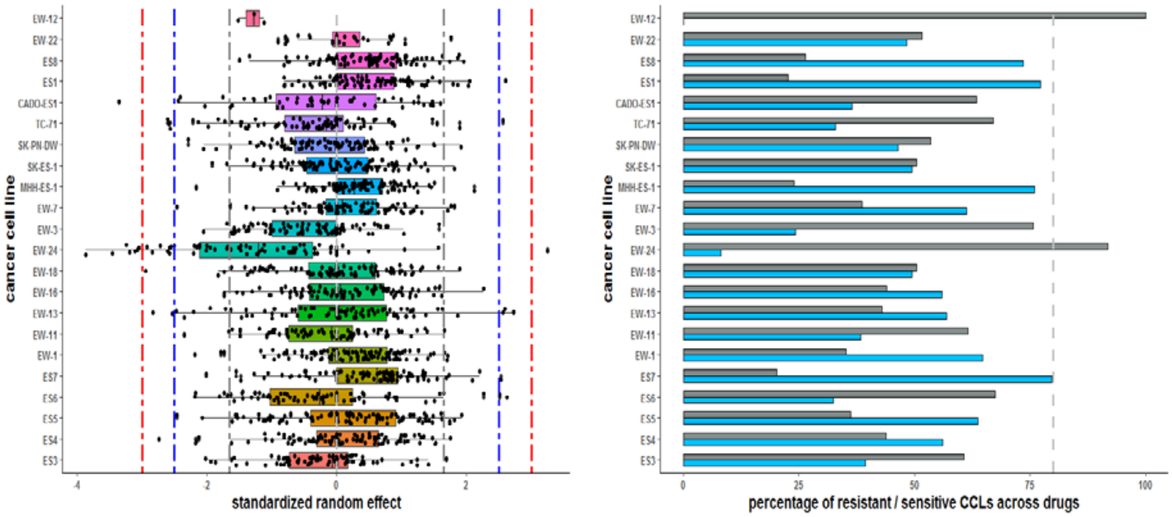

Glioma

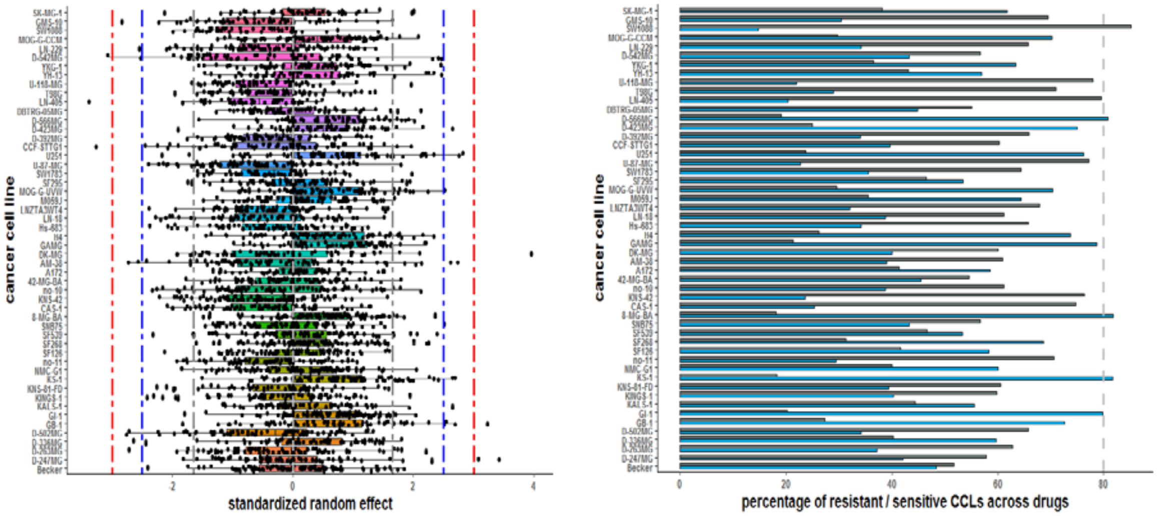

Head Neck

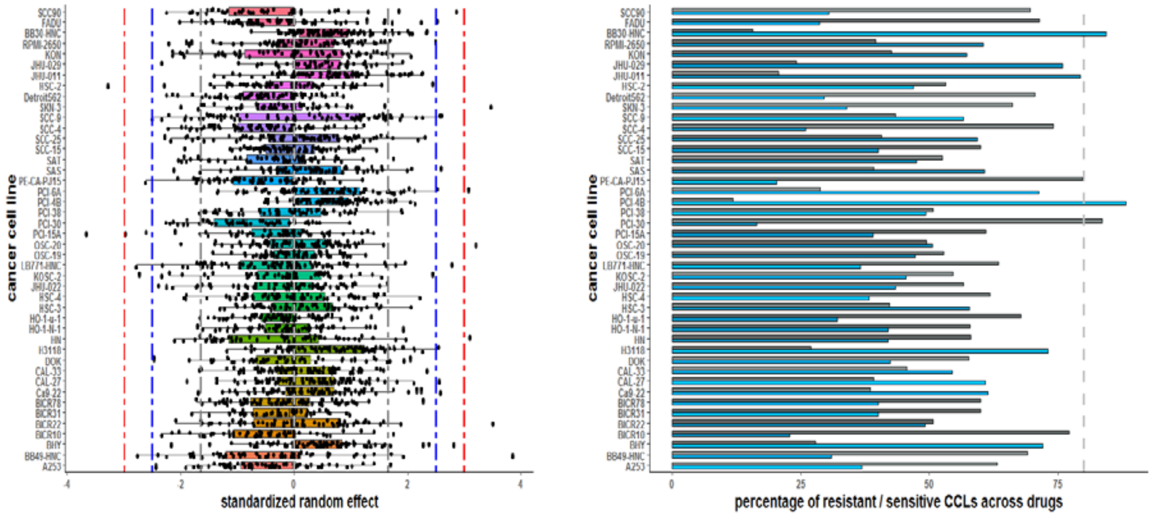

Kidney

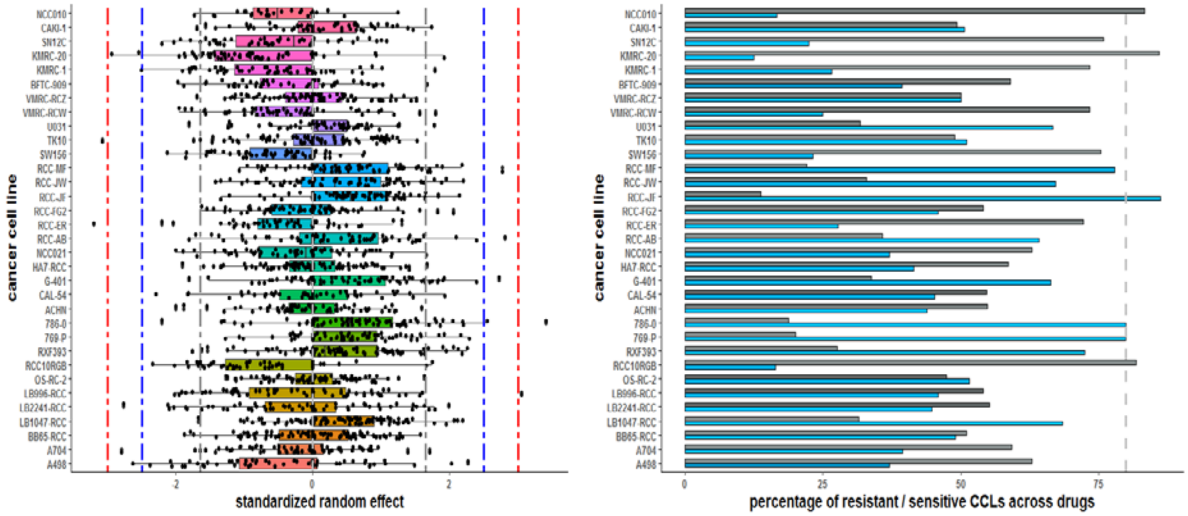

Large Intense

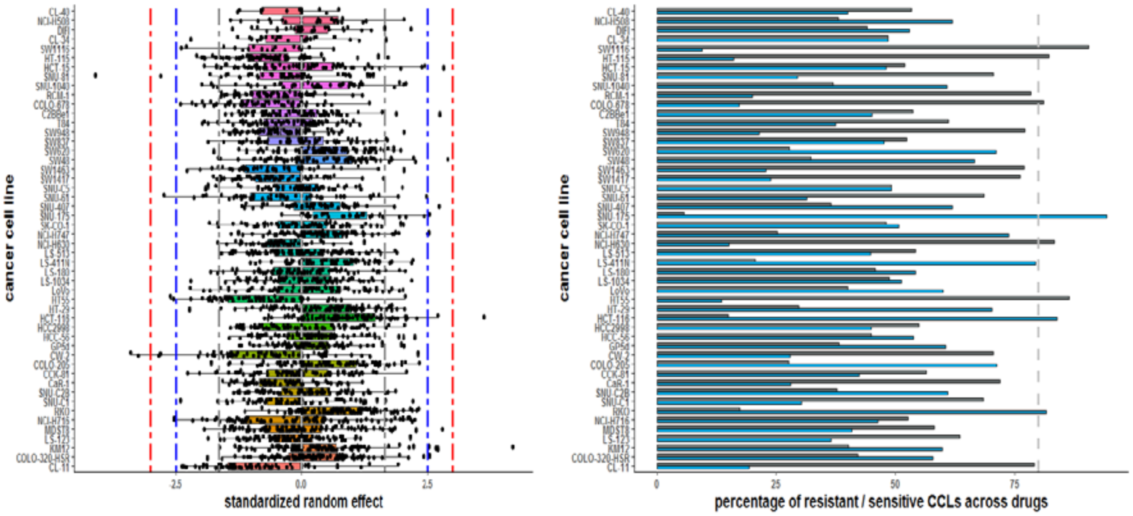

Liver

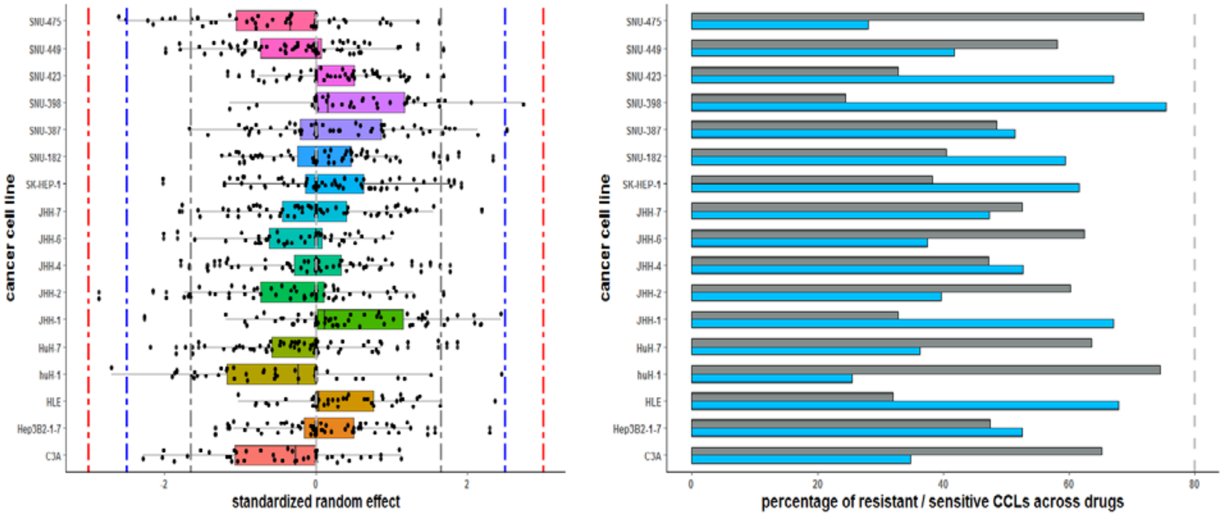

Lung NSCLC Adenocarcinoma

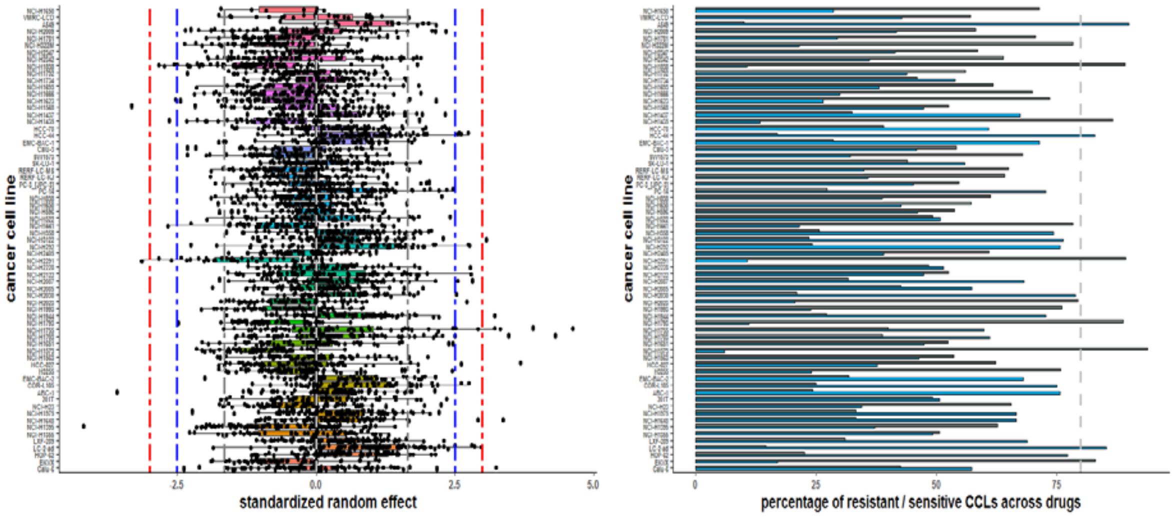

Lung NSCLC Large Cell

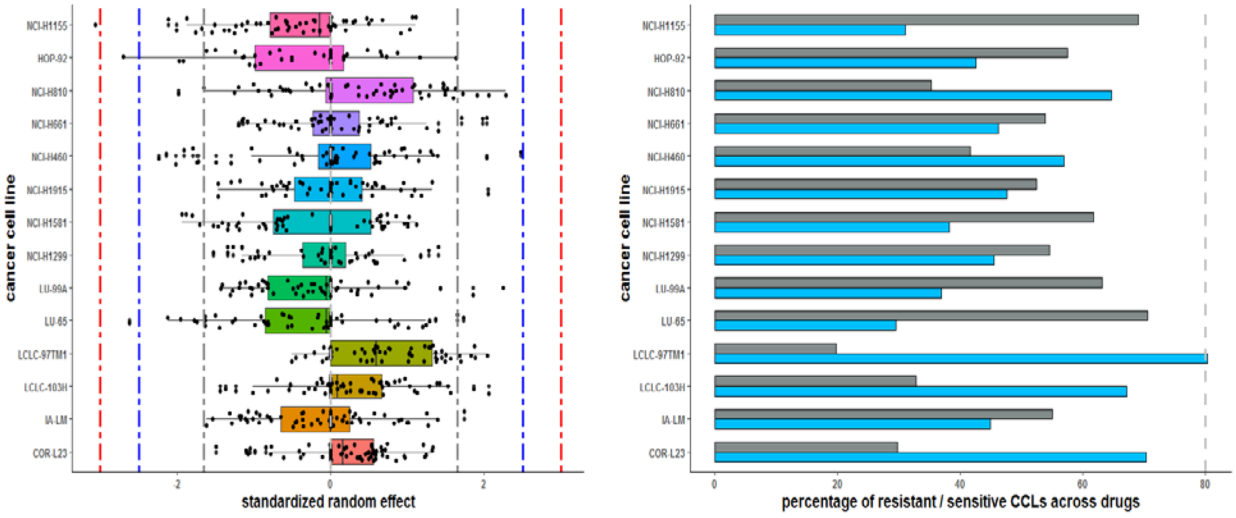

Lung NSCLC Squamous Cell Carcino

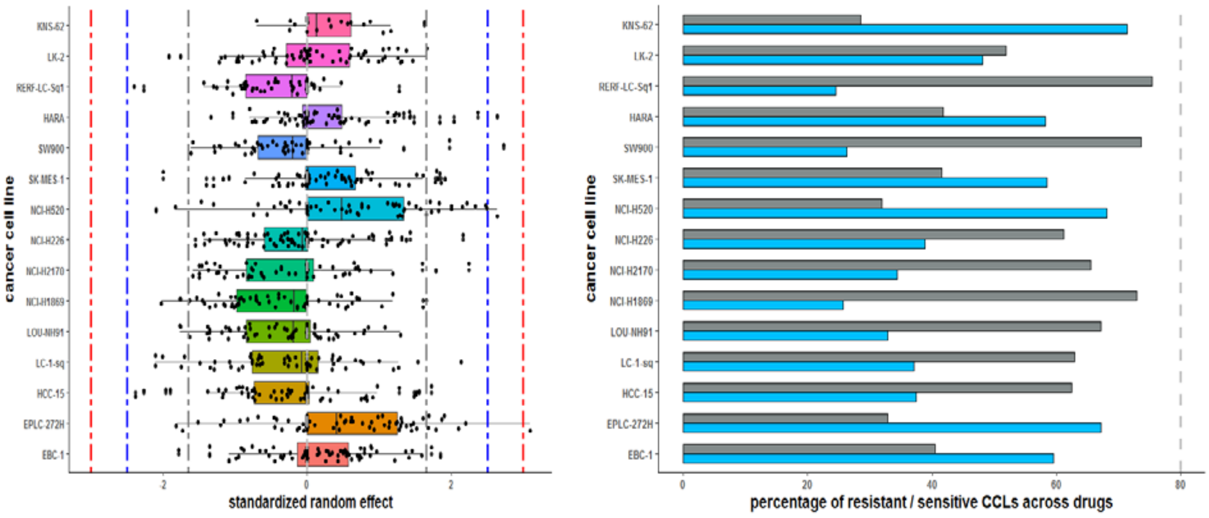

Lung Small Cell Carcinoma

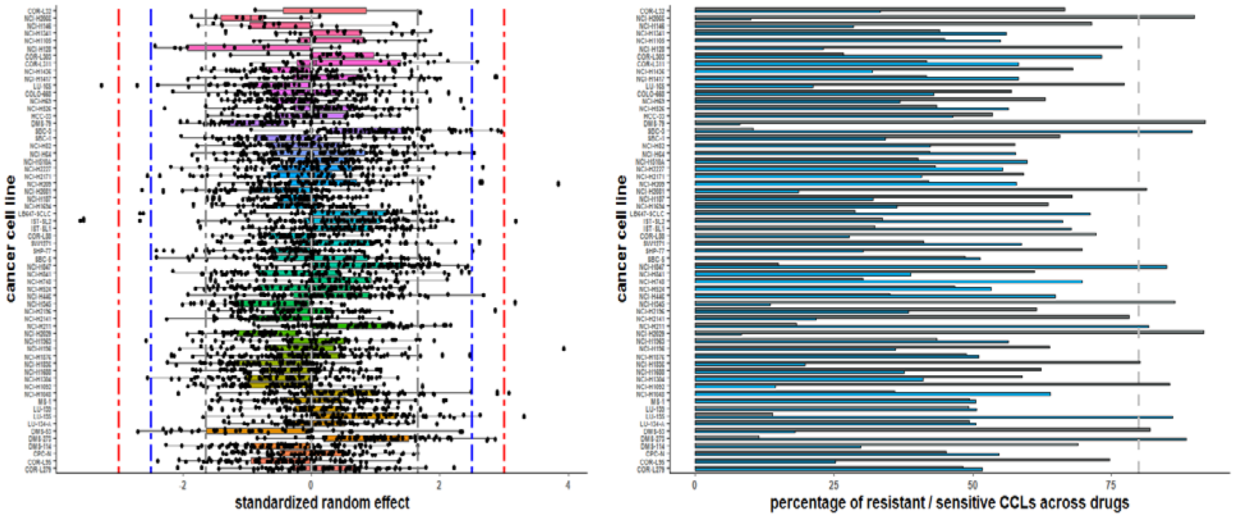

Lymphoblastic Leukemia

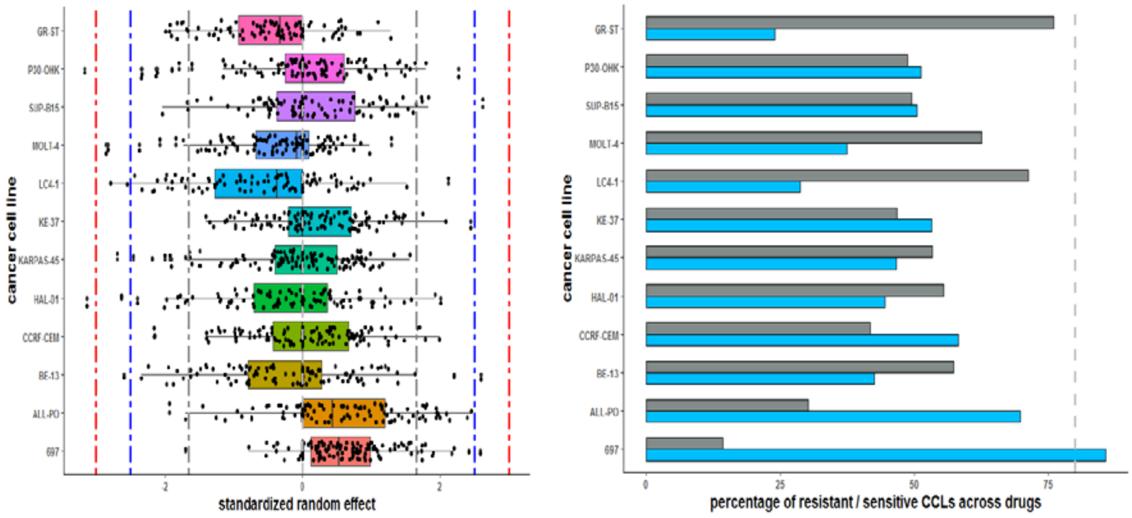

Lymphoid Neoplasm

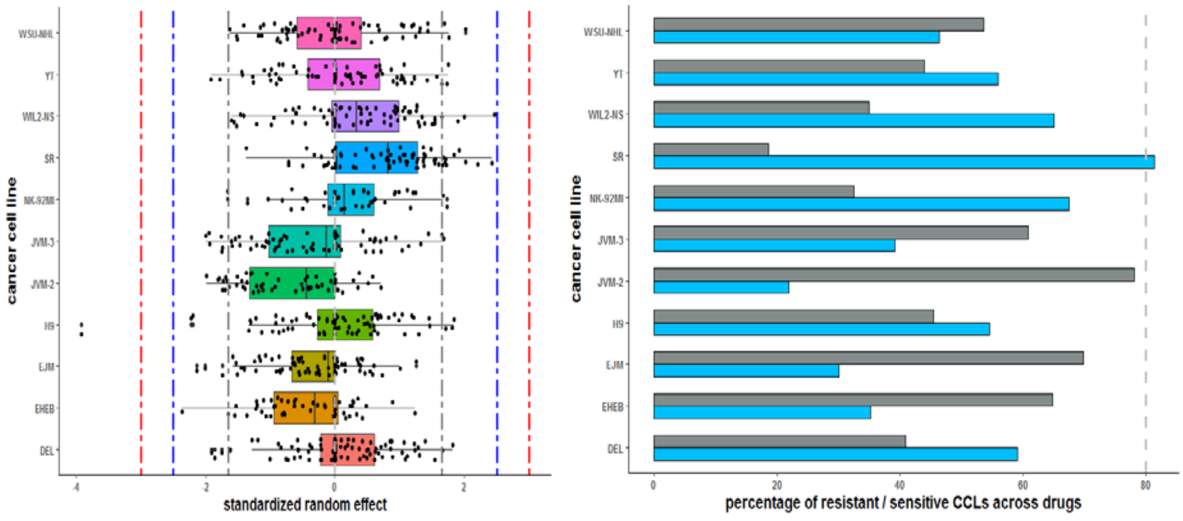

Melanoma

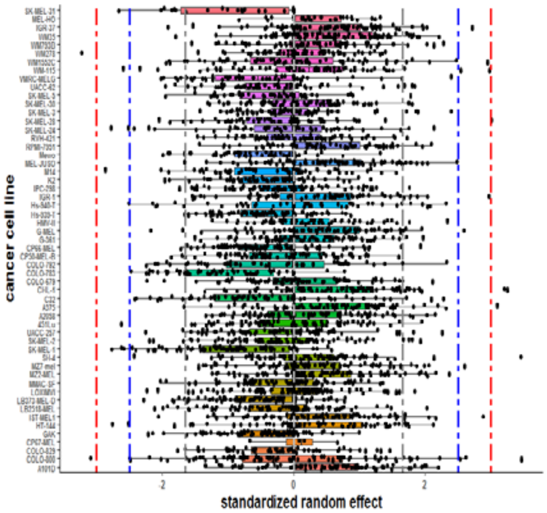

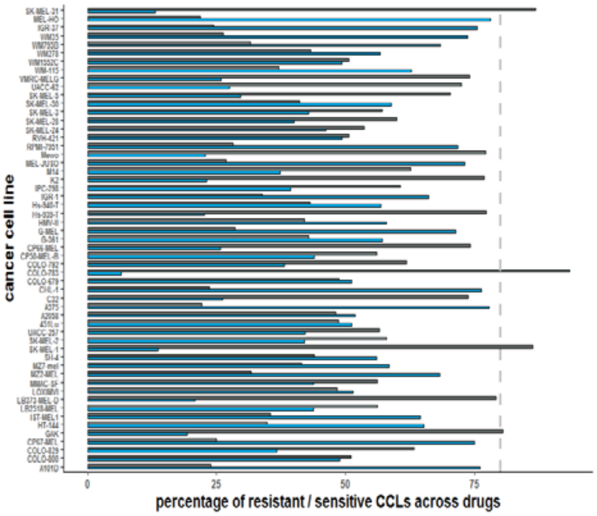

Mesothelioma

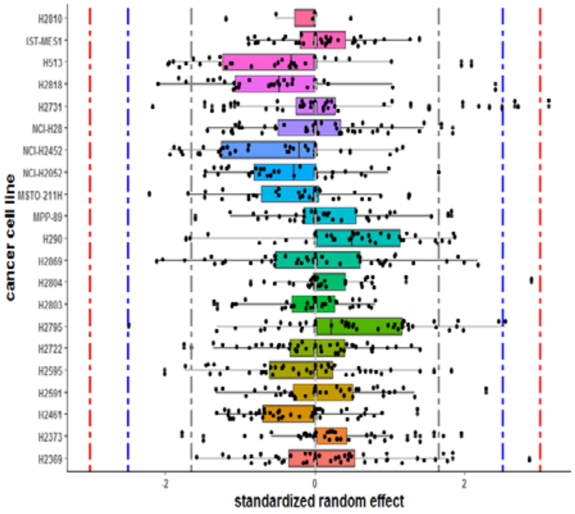

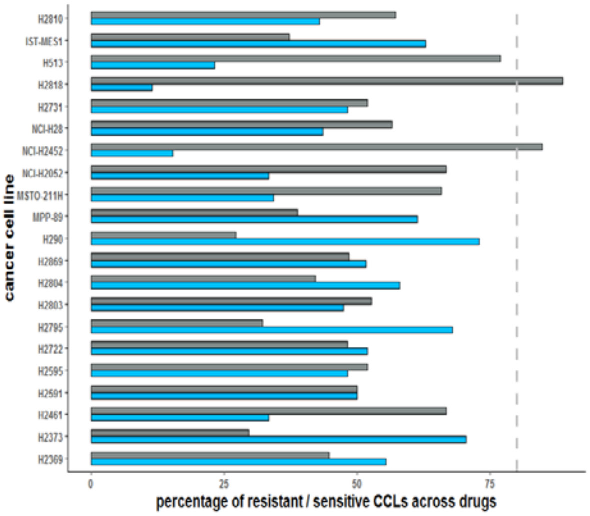

Myeloma

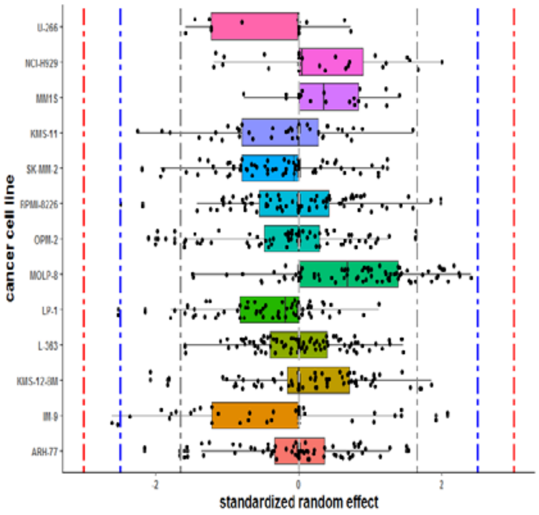

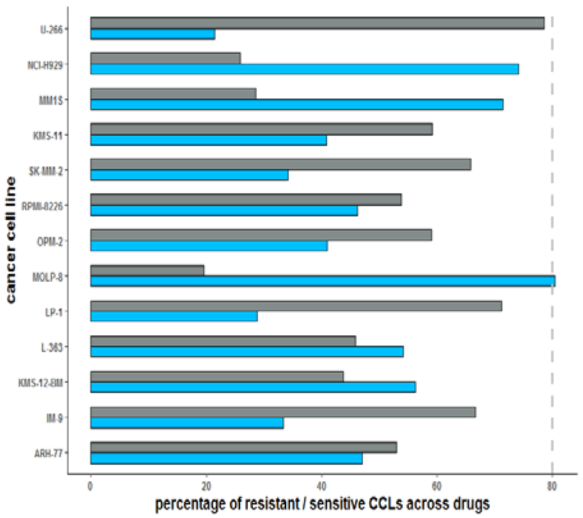

Neuroblastoma

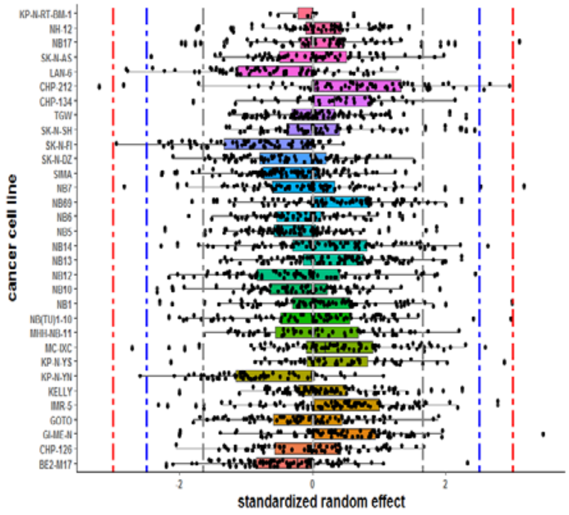

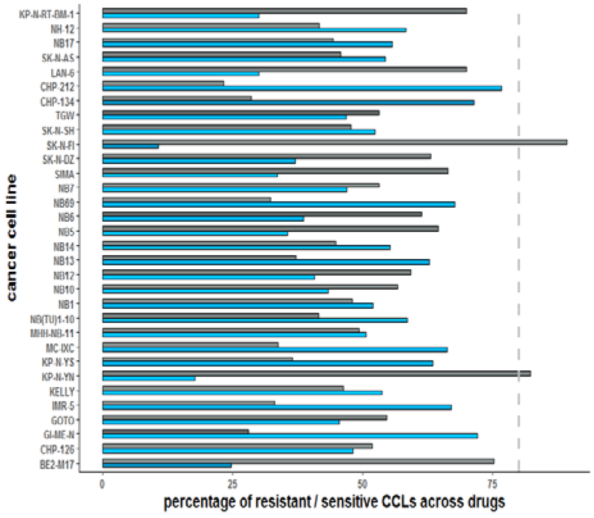

Oesophagus

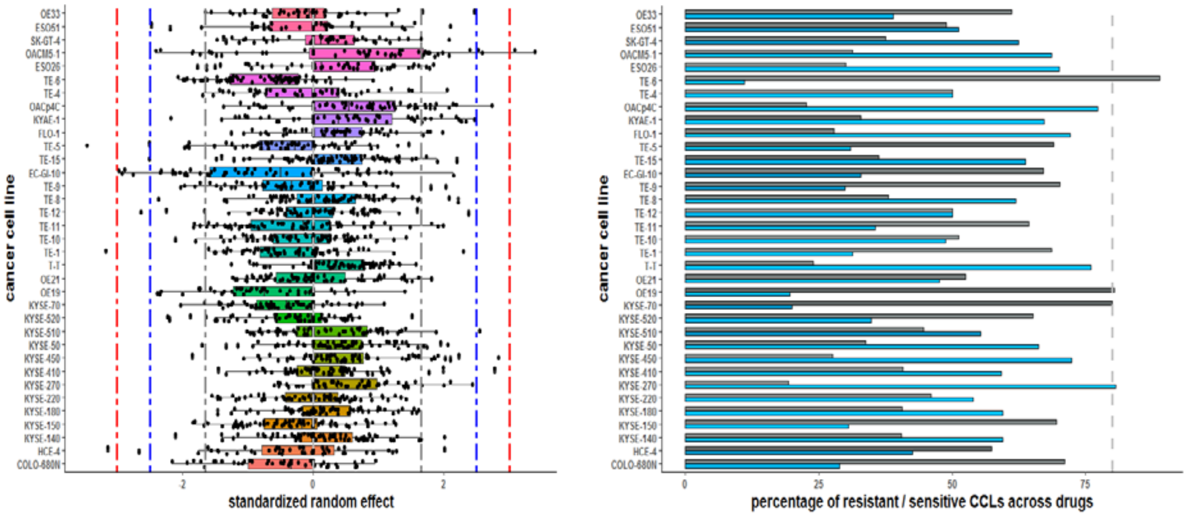

Ovary

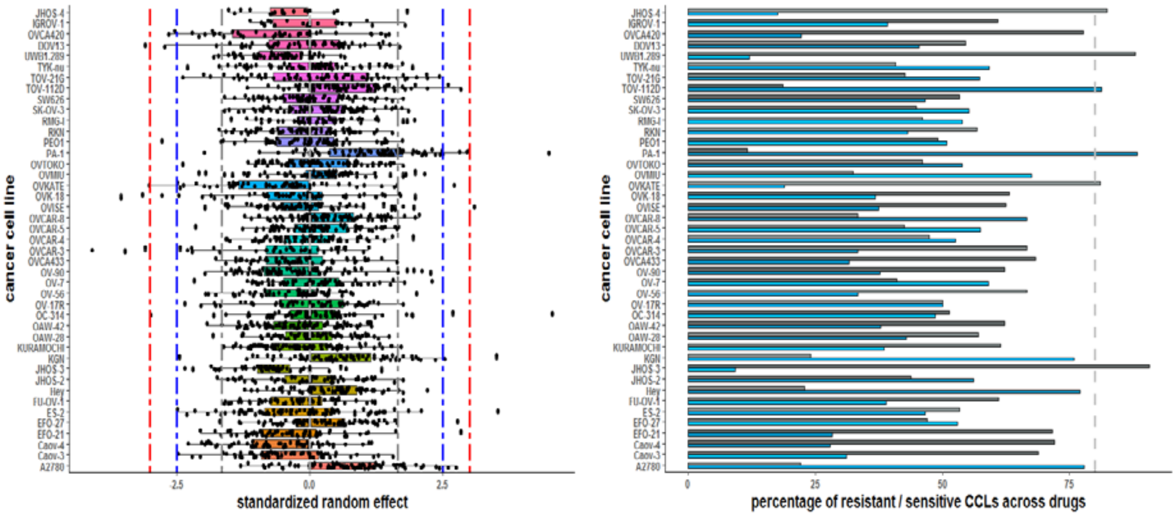

Pancreas

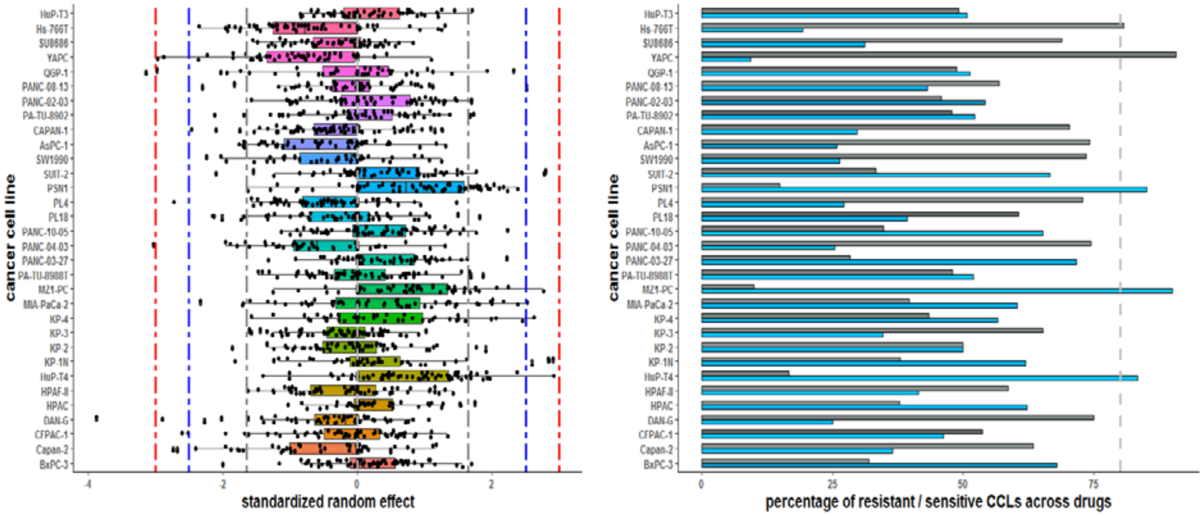

Stomach

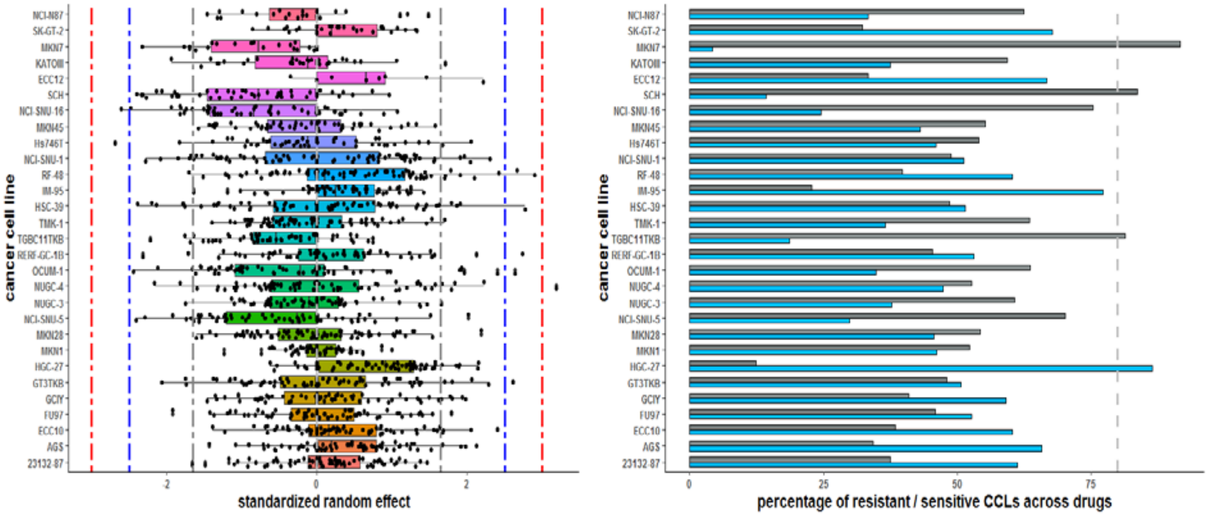

Thyroid

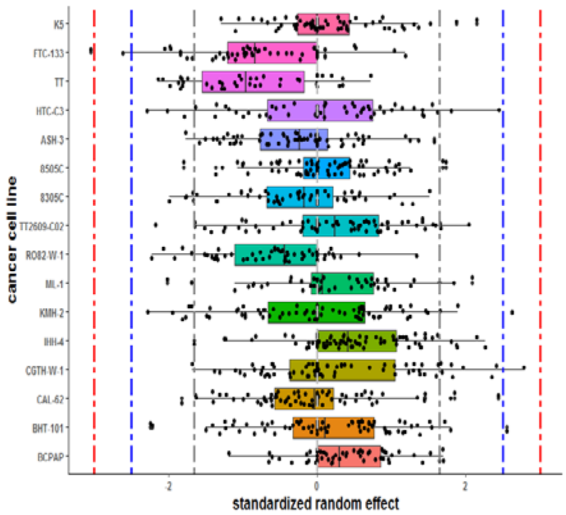

K5
FTC-133
TT
HTC-C3
ASH-3
8505C
8305C
TT2609-C02
RO82-W-1
ML-1
KMH-2
IHH-4
CGTH-W-1
CAL-62
BHT-101
BCPAP
cancer cell line
-2
0
2
standardized random effect

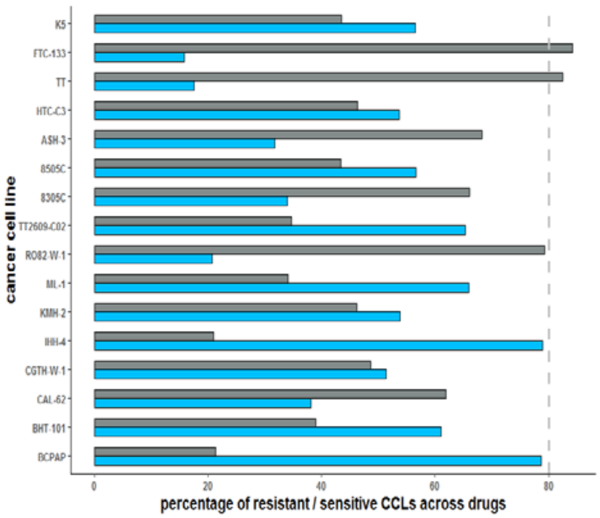

K5
FTC-133
TT
HTC-C3
ASH-3
8505C
8305C
TT2609-C02
RO82-W-1
ML-1
KMH-2
IHH-4
CGTH-W-1
CAL-62
BHT-101
BCPAP
cancer cell line
0
20
40
60
80
percentage of resistant / sensitive CCLs across drugs

Supplemental Figure 7: Compare estimates of EC50 for drug response data using the standardized random effect estimates (SREs) by fitting a NLME model (via *nlme* package) and nonlinear model (via *drc* package) for 354 CCLs and 15 drugs in both GDSC and CCLE. Scatter plots reporting the SREs for EC50 vs. the nonlinear estimate of EC50 for (**A**) CCLE and (**B**) GDSC. Bar plot representing the Spearman's rank correlation between SREs and nonlinear estimate of EC50 for (**C**) CCLE and (**D**) GDSC.

A.

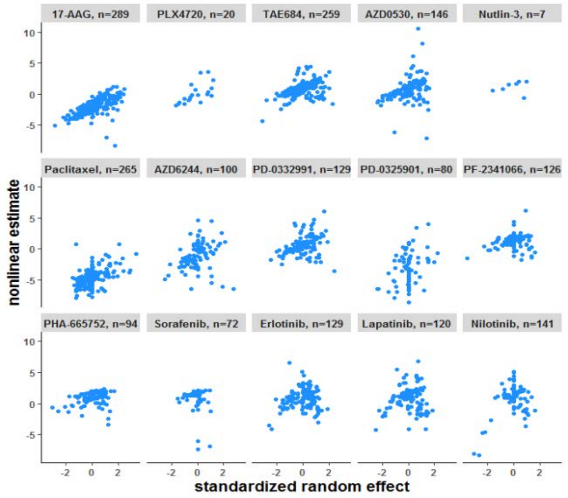

B.

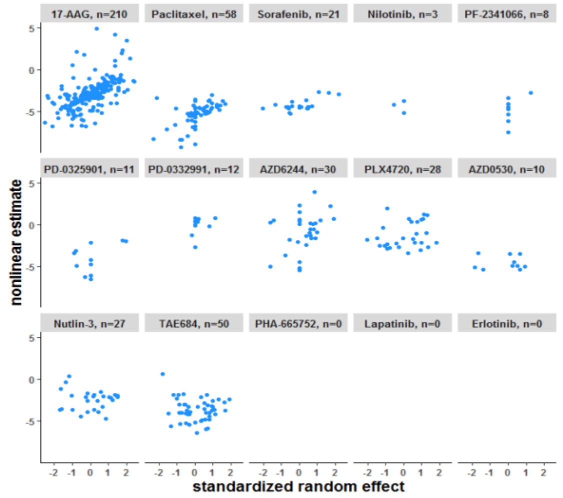

C.

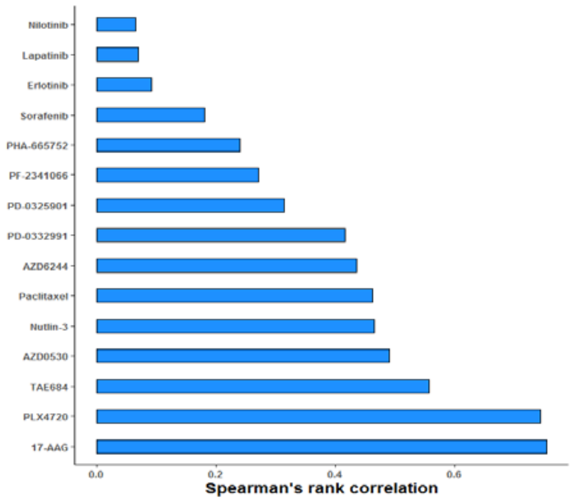

D.

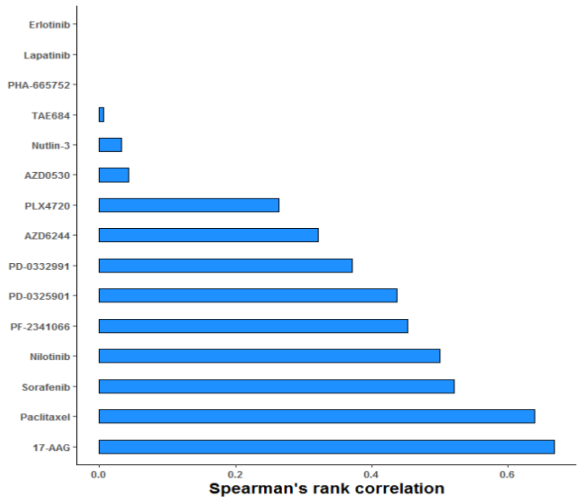

Supplemental Methods:

Assumption model checking is an essential step of statistical modeling that ensures the assumptions necessary for valid inference are upheld. In this study, due to the large number of drugs and cancer types across CCLE and GDSC studies, the model assumptions were not verified statistically for all models. However, model checking was completed for the models in which the outlier cell lines were determined (see **Tables 1** and **2**). For example, A2780 (ovary) was determined to be an outlier. Thus, we assessed the model assumptions for all ovarian CCLs included in the NLME analysis for a selected drug, here TKI258. In checking for model assumptions we looked at a histogram of the residuals, a normal qq-plot of the residuals, and a plot of the residuals against the fitted values. In general, we found the modeling assumptions to be valid for the majority of these selected drugs and cancer types, with a few drugs showing more "heavy tails" in the distribution than expected for a normal distribution. Below are the plots for the CCLE and GDSC selected cancer types and drugs.

CCLE

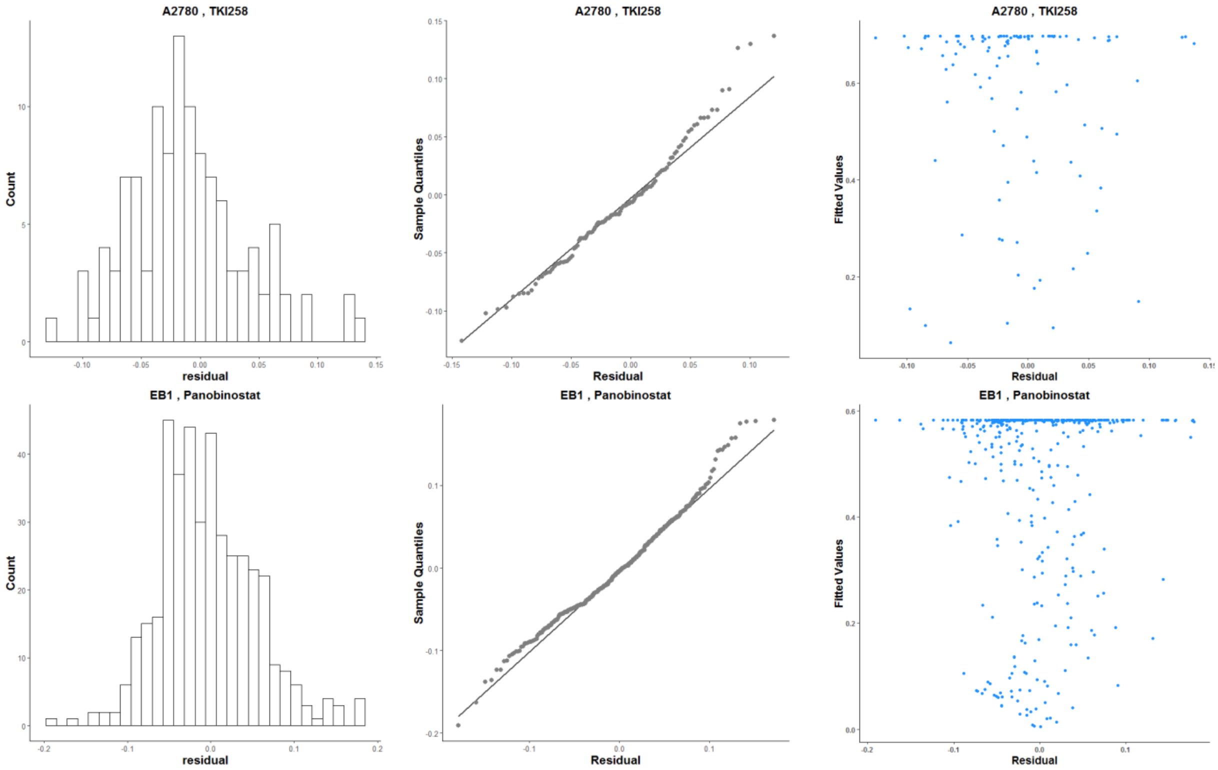

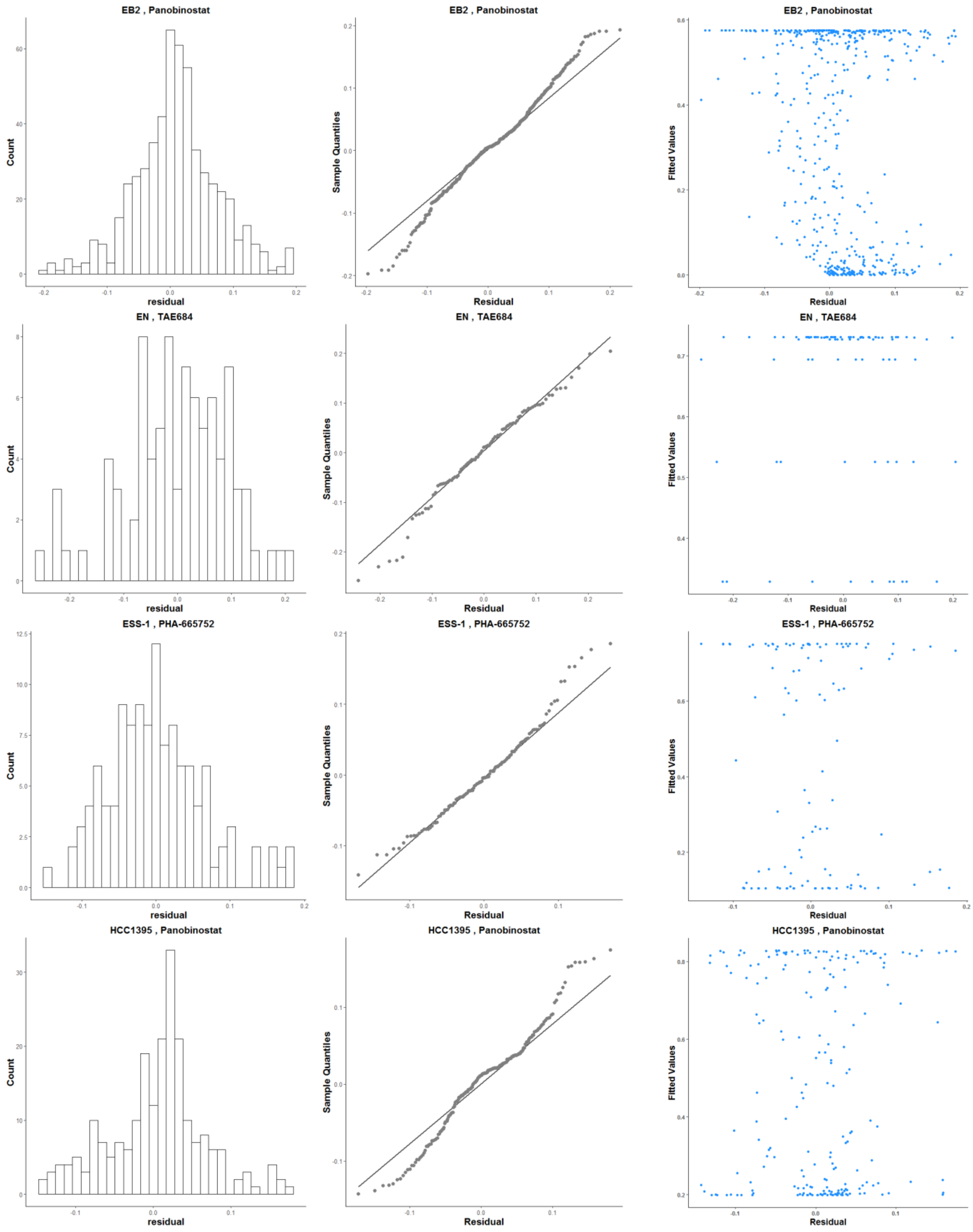

EB2 , Panobinostat
Count
residual
EB2 , Panobinostat
Sample Quantiles
Residual
EB2 , Panobinostat
Fitted Values
Residual
EN , TAE684
Count
residual
EN , TAE684
Sample Quantiles
Residual
EN , TAE684
Fitted Values
Residual
ESS-1 , PHA-665752
Count
residual
ESS-1 , PHA-665752
Sample Quantiles
Residual
ESS-1 , PHA-665752
Fitted Values
Residual
HCC1395 , Panobinostat
Count
residual
HCC1395 , Panobinostat
Sample Quantiles
Residual
HCC1395 , Panobinostat
Fitted Values
Residual

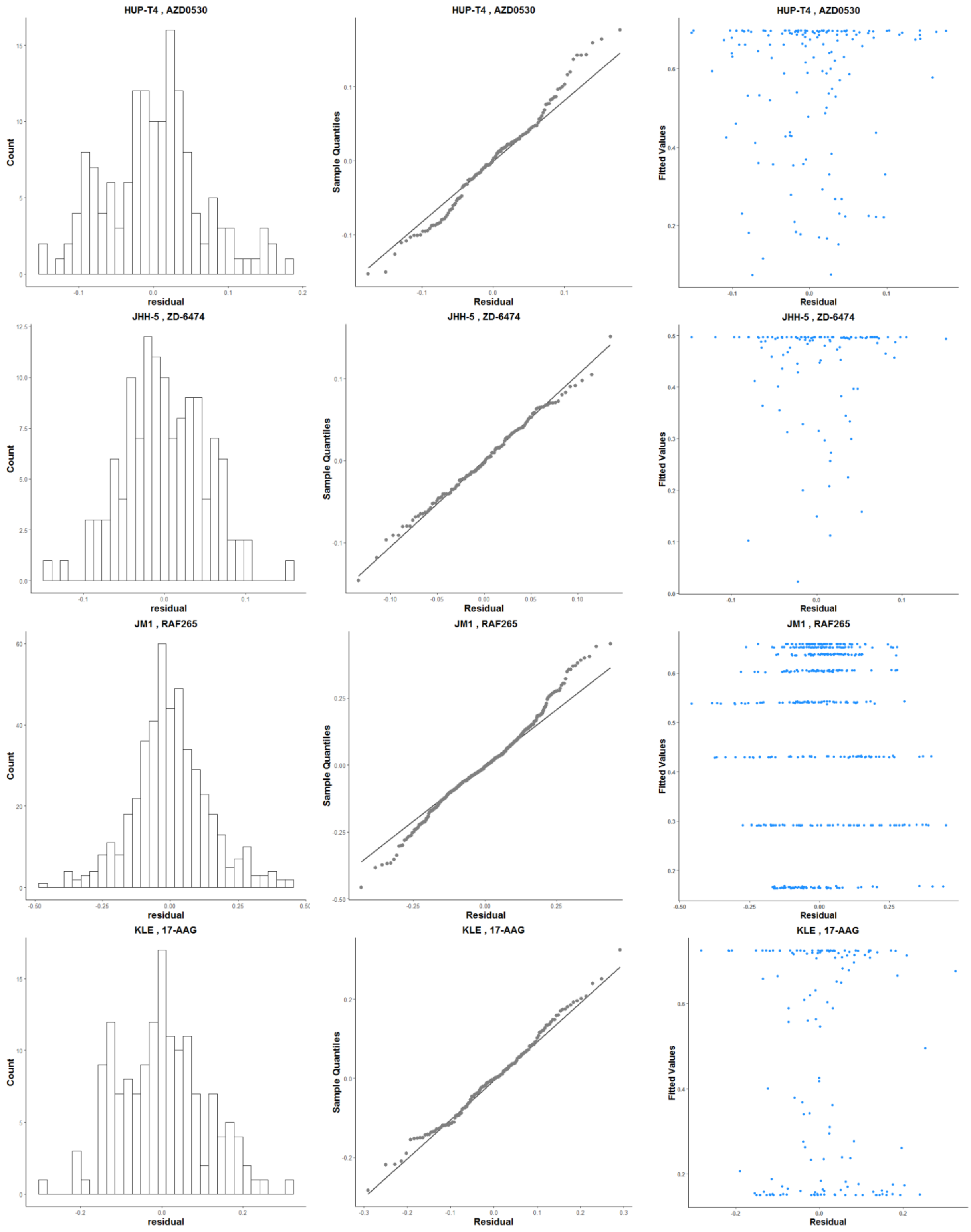

HUP-T4 , AZD0530
Count
residual
HUP-T4 , AZD0530
Sample Quantiles
Residual
HUP-T4 , AZD0530
Fitted Values
Residual
JHH-5 , ZD-6474
Count
residual
JHH-5 , ZD-6474
Sample Quantiles
Residual
JHH-5 , ZD-6474
Fitted Values
Residual
JM1 , RAF265
Count
residual
JM1 , RAF265
Sample Quantiles
Residual
JM1 , RAF265
Fitted Values
Residual
KLE , 17-AAG
Count
residual
KLE , 17-AAG
Sample Quantiles
Residual
KLE , 17-AAG
Fitted Values
Residual

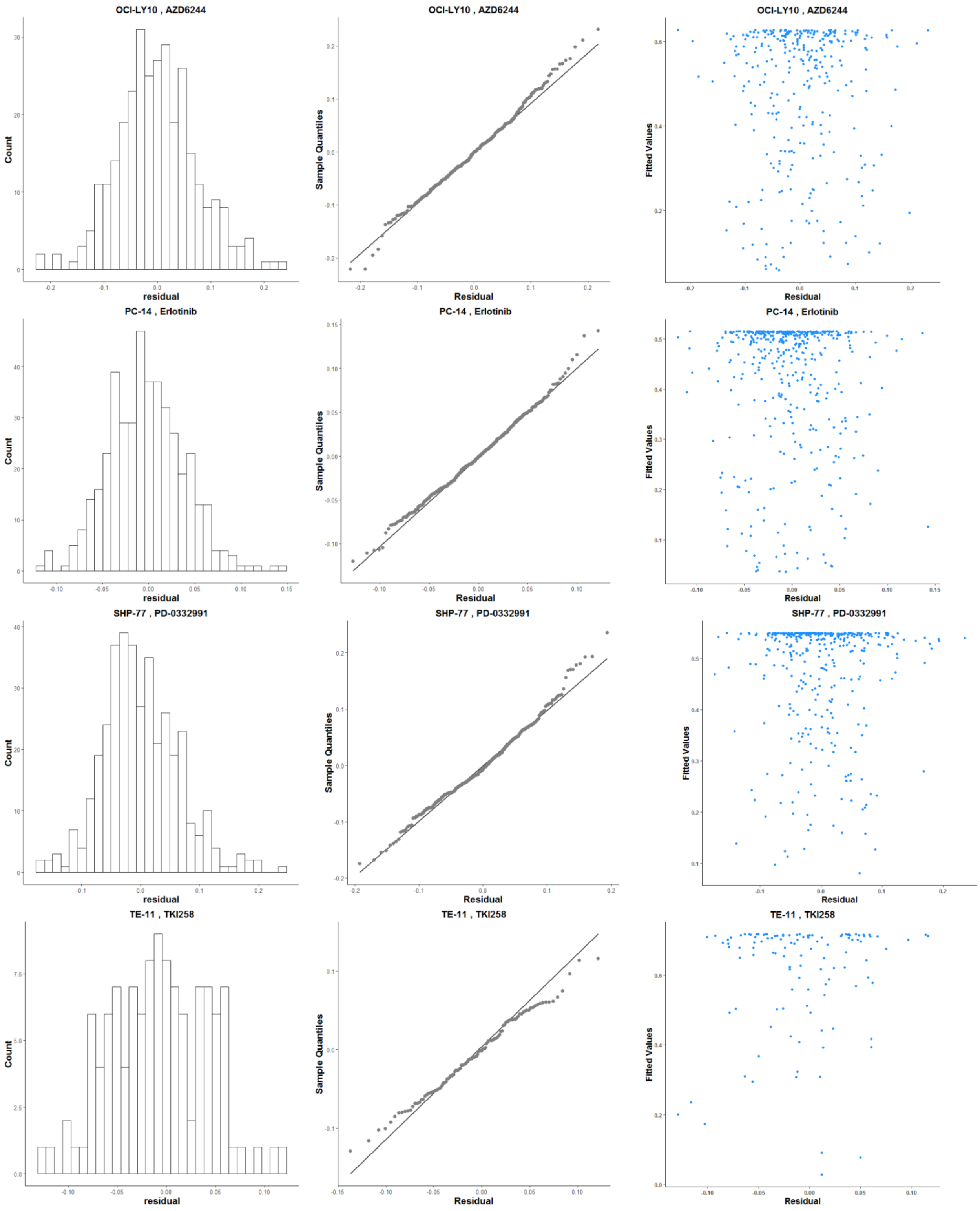
OCI-LY10 , AZD6244
Count
residual
OCI-LY10 , AZD6244
Sample Quantiles
Residual
OCI-LY10 , AZD6244
Fitted Values
Residual
PC-14 , Erlotinib
Count
residual
PC-14 , Erlotinib
Sample Quantiles
Residual
PC-14 , Erlotinib
Fitted Values
Residual
SHP-77 , PD-0332991
Count
residual
SHP-77 , PD-0332991
Sample Quantiles
Residual
SHP-77 , PD-0332991
Fitted Values
Residual
TE-11 , TKI258
Count
residual
TE-11 , TKI258
Sample Quantiles
Residual
TE-11 , TKI258
Fitted Values
Residual

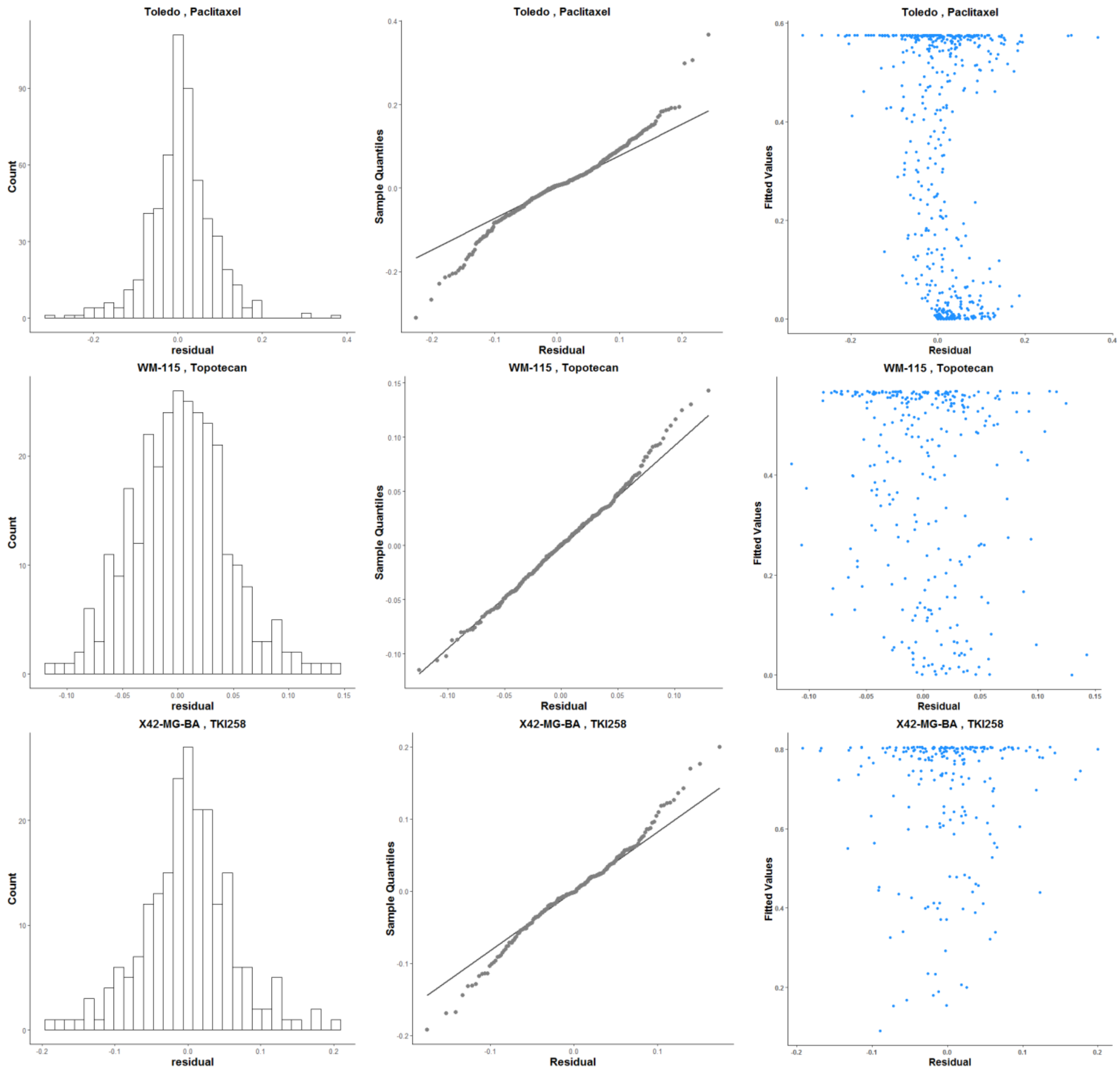
Toledo , Paclitaxel
Count
residual
Toledo , Paclitaxel
Sample Quantiles
Residual
Toledo , Paclitaxel
Fitted Values
Residual
WM-115 , Topotecan
Count
residual
WM-115 , Topotecan
Sample Quantiles
Residual
WM-115 , Topotecan
Fitted Values
Residual
X42-MG-BA , TKI258
Count
residual
X42-MG-BA , TKI258
Sample Quantiles
Residual
X42-MG-BA , TKI258
Fitted Values
Residual

GDSC

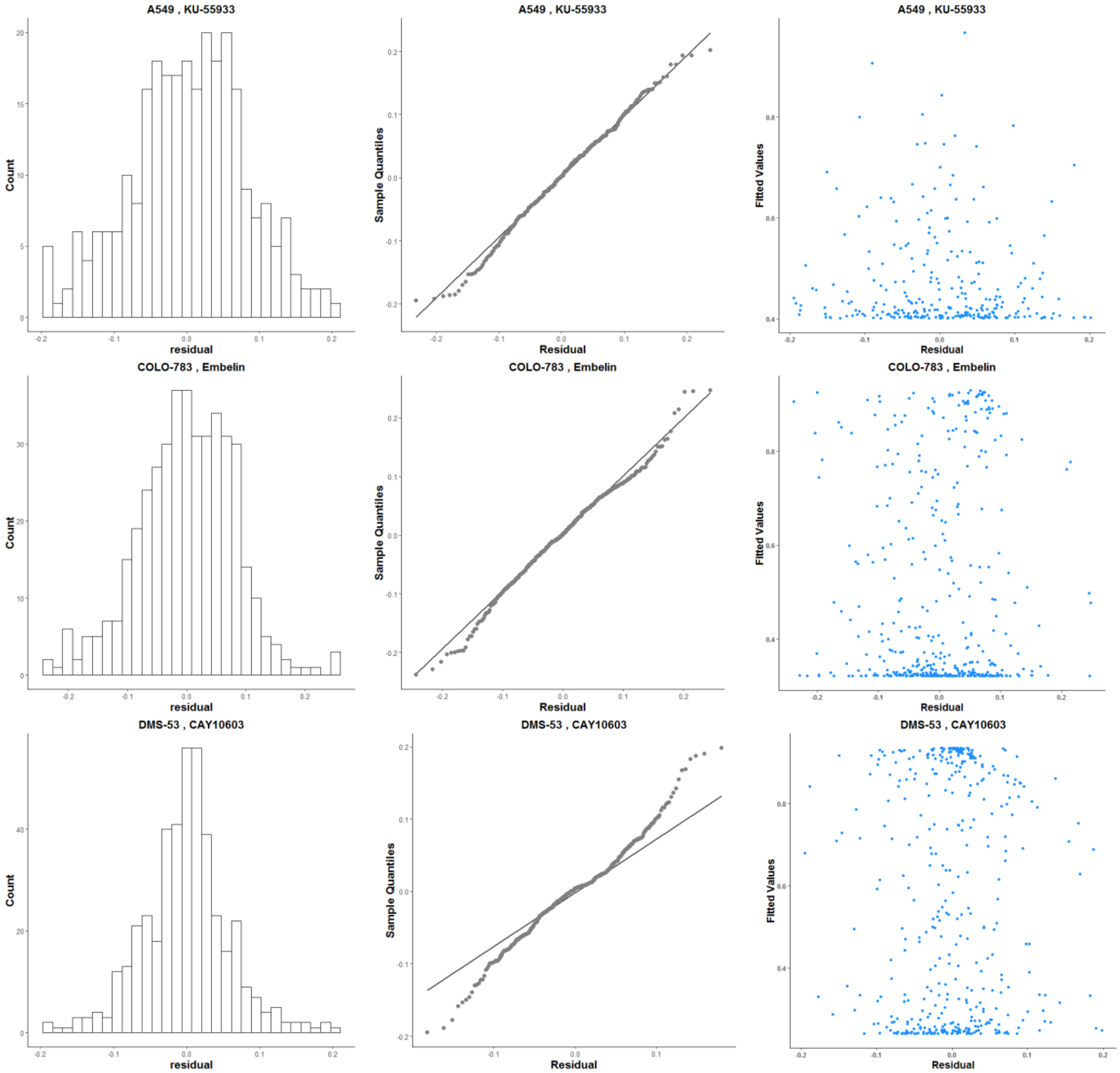

A549 , KU-55933
Count
residual
Sample Quantiles
Residual
Fitted Values
COLO-783 , Embelin
DMS-53 , CAY10603

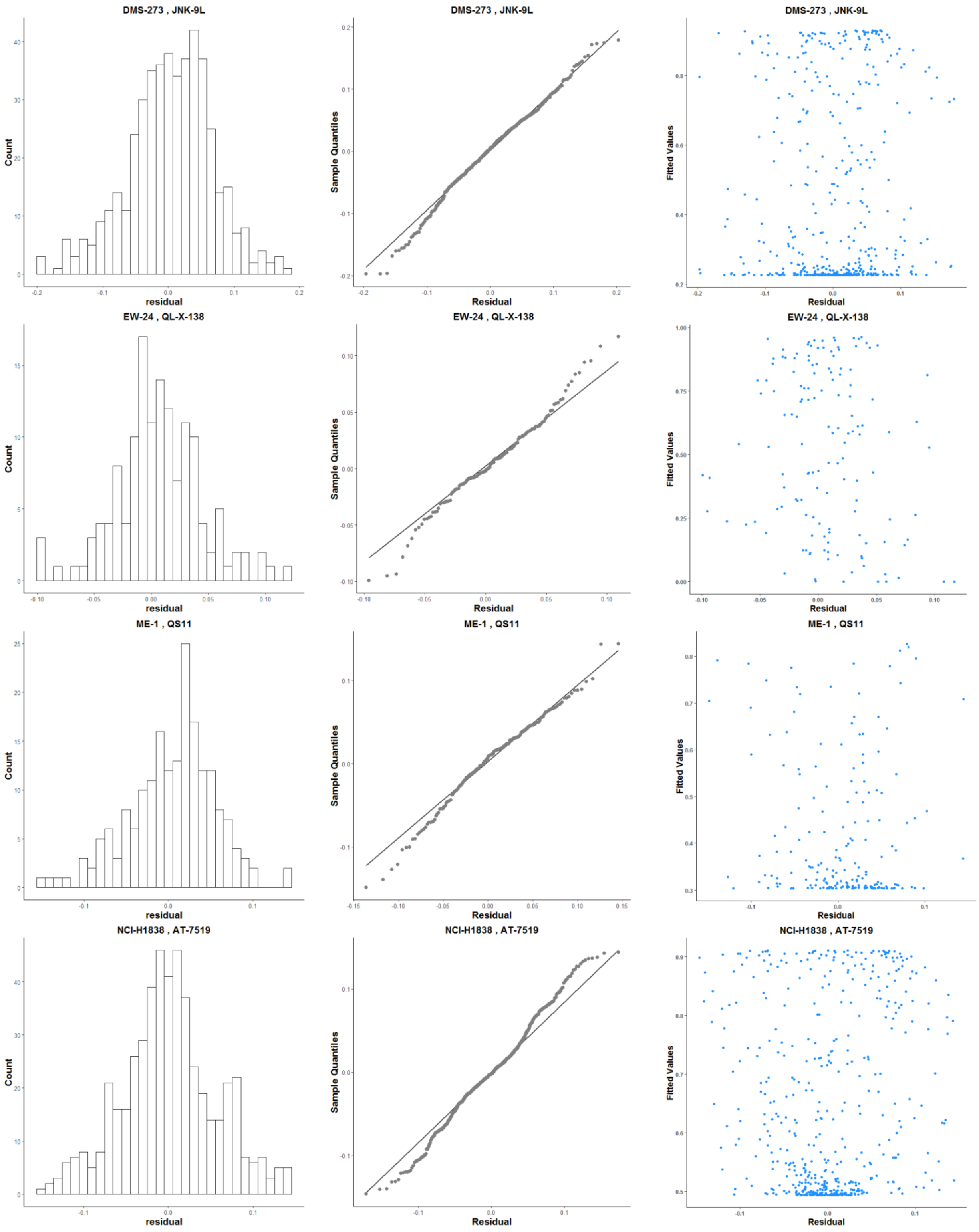
DMS-273 , JNK-9L
Count
residual
Sample Quantiles
Residual
Fitted Values
EW-24 , QL-X-138
ME-1 , QS11
NCI-H1838 , AT-7519

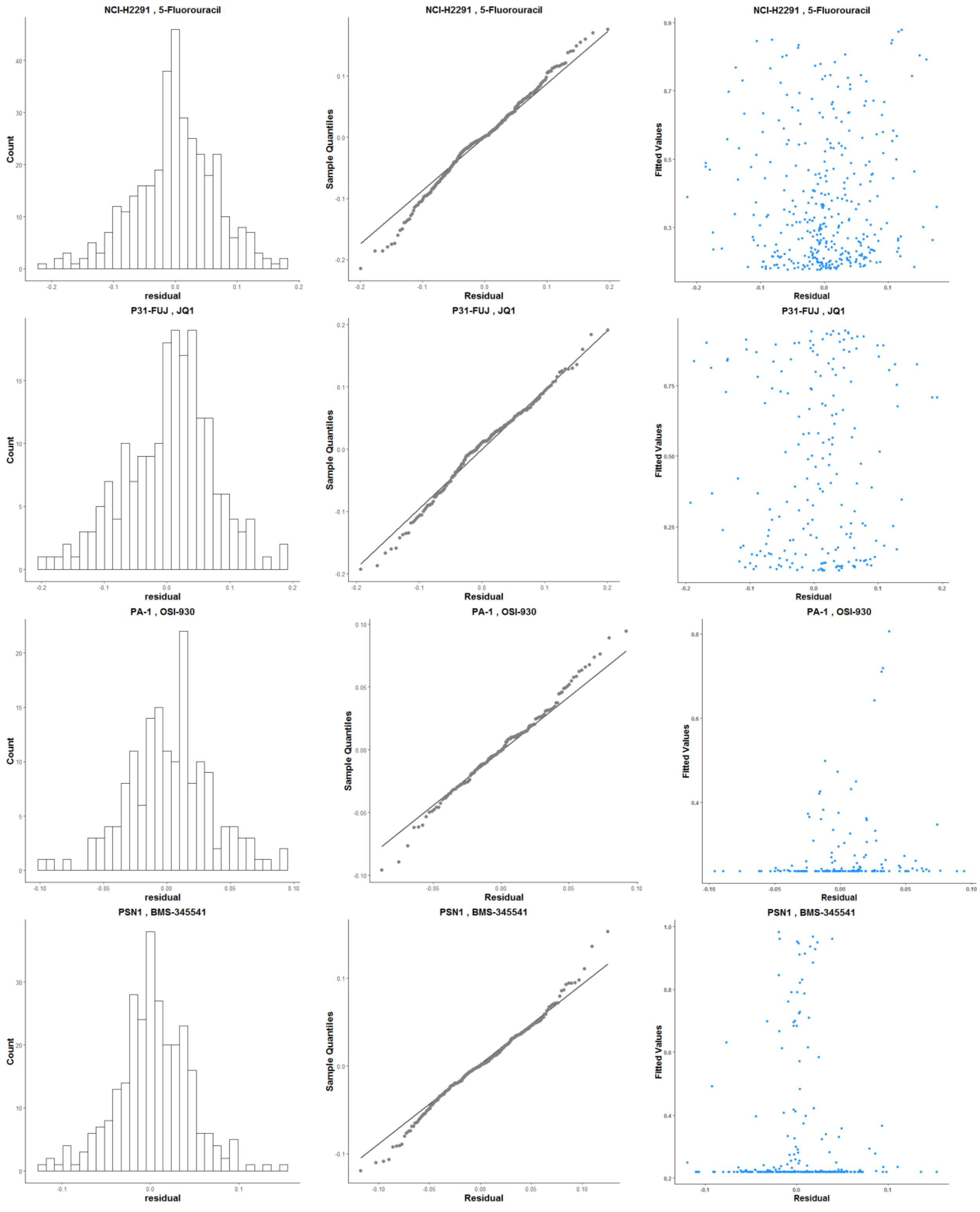
NCI-H2291 , 5-Fluorouracil
Count
residual
NCI-H2291 , 5-Fluorouracil
Sample Quantiles
Residual
NCI-H2291 , 5-Fluorouracil
Fitted Values
Residual
P31-FUJ , JQ1
Count
residual
P31-FUJ , JQ1
Sample Quantiles
Residual
P31-FUJ , JQ1
Fitted Values
Residual
PA-1 , OSI-930
Count
residual
PA-1 , OSI-930
Sample Quantiles
Residual
PA-1 , OSI-930
Fitted Values
Residual
PSN1 , BMS-345541
Count
residual
PSN1 , BMS-345541
Sample Quantiles
Residual
PSN1 , BMS-345541
Fitted Values
Residual

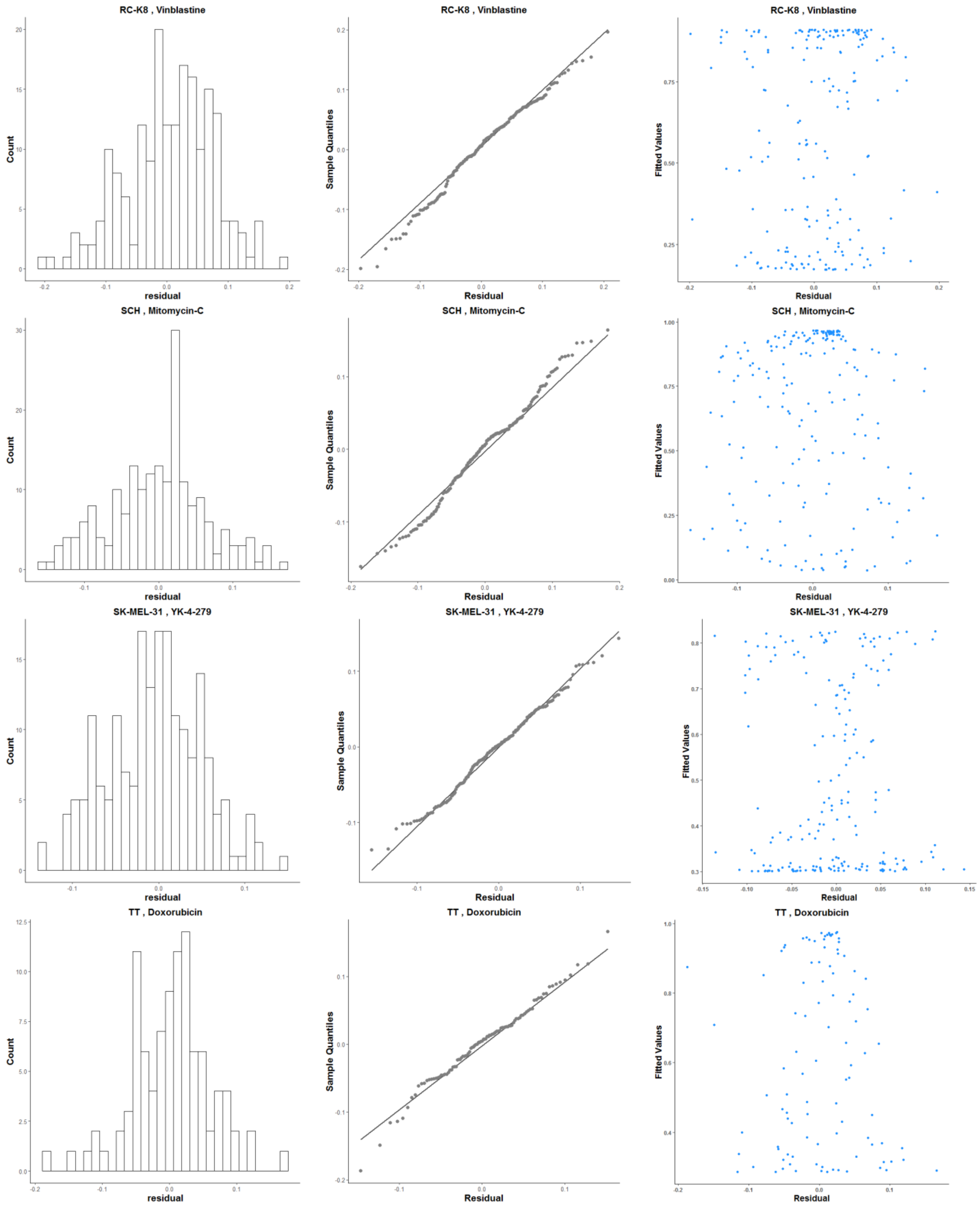
RC-K8 , Vinblastine
Count
residual
RC-K8 , Vinblastine
Sample Quantiles
Residual
RC-K8 , Vinblastine
Fitted Values
Residual
SCH , Mitomycin-C
Count
residual
SCH , Mitomycin-C
Sample Quantiles
Residual
SCH , Mitomycin-C
Fitted Values
Residual
SK-MEL-31 , YK-4-279
Count
residual
SK-MEL-31 , YK-4-279
Sample Quantiles
Residual
SK-MEL-31 , YK-4-279
Fitted Values
Residual
TT , Doxorubicin
Count
residual
TT , Doxorubicin
Sample Quantiles
Residual
TT , Doxorubicin
Fitted Values
Residual
